# Biochemical and agronomic responses of guar to nitrogen, *Bradyrhizobium* inoculation and deficit irrigation under arid and semi-arid conditions

**DOI:** 10.1038/s41598-026-52375-0

**Published:** 2026-05-11

**Authors:** Gholamali Akbari, Saeed Norouzi, Iraj Alahdadi, Elias Soltani, Mohammadali Norouzian

**Affiliations:** 1 Department of Agronomy and Plant Breeding Sciences, Aburaihan Campus, University of Tehran, Pakdasht, Tehran Iran; 2 Department of Animal and Poultry Sciences, Aburaihan Campus, University of Tehran, Pakdasht, Tehran Iran

**Keywords:** *Bradyrhizobium japonicum*, Drought stress, Enzymatic/non-enzymatic activities, Forage quality, Grain protein, Grain yield, Metabolizable energy, Physiology, Plant sciences

## Abstract

The present study aimed to investigate the effects of various levels of chemical nitrogen fertilizer and inoculation with different strains of *Bradyrhizobium japonicum* on several phenological, quantitative, qualitative, and fodder-related characteristics of guar (*Cyamopsis tetragonoloba* L.) under varying water-deficit regimes. A two-year field experiment (2021–2022) was conducted as a split-factorial arrangement based on a randomized complete block design with three replications to evaluate the interactive effects of nitrogen fertilization (0, 50, and 100 kg ha^− 1^) and seed inoculation with strains RS-150 and RS-153 under different irrigation regimes. Deficit irrigation significantly reduced grain yield, while full irrigation × 100 kg N ha^− 1^ yielded the highest GY (3658.2 kg ha^− 1^) and severe deficit × no N applicatopn the lowest (1017.8 kg ha^− 1^). Application of 100 kg N ha^− 1^ increased GY by 30.99% compared with the control. Inoculation with RS-153 enhanced grain number per m² and 1000-grain weight by 14.41% and 14.54%, respectively, relative to non-inoculated plants. Full irrigation also increased grain protein content by up to 22.19% compared with severe deficit. Soluble sugars accumulated under water stress, reaching 490.16 µg g^− 1^ DW under deficit irrigation × 100 kg N ha^− 1^ × RS-150, indicating osmotic adjustment. Crude protein, metabolizable energy, and dry matter digestibility were further improved under full irrigation and higher nitrogen supply. Overall, integrated nitrogen management and rhizobial inoculation partially mitigated drought-induced yield reductions and improved the forage quality of guar under semi-arid conditions.

##  Introduction

Agricultural production has long served as a primary source of livelihood and a key contributor to feeding the growing global population^1,2^. However, farmland degradation remains a major constraint on crop productivity, emphasizing the importance of appropriate management practices to sustain soil fertility and yield^3,4^. Effective resource and nutrient management, particularly of nitrogen (N), one of the most limiting nutrients, plays a crucial role in determining crop growth and yield quality^5–7^. In Iran, where about 88% of the land area is arid or semi-arid, soils are typically poor in organic matter and N content^8,9^. Although chemical N fertilizers can enhance plant growth by improving root development, leaf area, and nutrient uptake^10,11^, their excessive use accelerates soil organic matter depletion, acidification, soil erosion, and environmental pollution^12,13^. In general, integrating organic manures with conventional fertilization improves soil carbon and nitrogen stocks while sustaining yield^14^. In this regard, legumes, as efficient atmospheric N fixers, enrich soil fertility and reduce dependence on chemical fertilizers, representing a sustainable approach to improving soil nutrient status^15–18^. Guar (*Cyamopsis tetragonoloba* L.), an annual drought- and heat-tolerant legume native to India and Pakistan, thrives in sandy and well-drained clay soils and serves diverse purposes, including fodder, food, and industrial use^19–21^. Its rich composition of galactomannan, phenolic, and flavonoid compounds imparts antibacterial and medicinal properties, with traditional applications for treating diabetes, asthma, anemia, and inflammation^22–26^.

Another approach toward the sustainable intensification of agriculture to improve soil fertility and plant growth is the use of plant growth-promoting rhizobacteria (PGPRs) or bio-fertilizers^27,28^. PGPRs are eco-friendly alternatives to chemical fertilizers, and their application began about 60 years ago^29,30^. These microorganisms enhance vegetative growth, nutrient uptake, nutrient solubility, and siderophore production, as well as synthesize phytohormones, enzymes, and metabolites involved in plant growth and stress tolerance, particularly under drought conditions^30–34^. Well-known PGPRs include *Rhizobium*, *Bradyrhizobium*, *Bacillus*, *Azospirillum*, *Azotobacter*, *Acinetobacter*, *Pseudomonas*, *Mycorrhiza*, and *Trichoderma*^35–37^. Inoculation of legumes with *Rhizobium* strains has been reported to improve growth traits such as shoot biomass, plant height, branch numbers, nodules, pods, grain weight, yield, and protein content^38^. Although these bacteria generally promote plant growth and yield, their symbiotic efficiency in N fixation is influenced by environmental factors such as water availability, soil salinity, pH, nutrient deficiency, ion toxicity, and temperature extremes^39–42^, which can exert either beneficial or adverse effects on plants^43^. Furthermore, bio-fertilizers can reduce the need for chemical N fertilizers, convert N compounds into amino acids, improve nutrient availability, root development, seed germination, and overall plant growth^44–46^.

Plants are constantly exposed to various biotic and abiotic stresses under diverse environmental conditions^47,48^. Among these, water availability is a major determinant of plant growth and productivity^49^. Drought stress, particularly in arid and semi-arid regions such as Iran, is one of the most limiting factors for plant performance and yield^50–52^. Developing efficient irrigation systems remains a major challenge, as water requirements vary with growth stage, plant sensitivity, climatic conditions, and irrigation intervals^53–55^. Water stress restricts evapotranspiration via stomatal closure, limits carbon assimilation, biomass accumulation, photosynthetic activities, and ultimately reduces grain yield^56–59^. It also promotes excessive formation of reactive oxygen species (ROS), including hydrogen peroxide and superoxide radicals, leading to oxidative damage, reduced photosynthetic and water-use efficiency, lipid peroxidation, and cell death^60–62^. To counter these effects, plants enhance their defense mechanisms through osmolyte accumulation (e.g., proline and soluble sugars), antioxidant enzyme activation, and synthesis of secondary metabolites (e.g., flavonoids and phenolics), increased abscisic acid, and maintenance of leaf water status^63^. In guar, supplemental irrigation improves plant height, pod number, biomass, and grain yield, while full irrigation enhances protein and galactomannan content^64^. Conversely, drought cycles markedly reduce yield components and water use efficiency^65–67^. Despite these insights, limited information exists on how integrated management of chemical and biological nitrogen sources under deficit irrigation can improve guar performance. Furthermore, water-deficit stress, besides its negative impact on crop yield, induces significant metabolic changes and accumulation of secondary metabolites in plants, while nitrogen application can enhance drought tolerance and water use efficiency^59,68^, partially mitigating these stress-induced metabolic effects. Researchers also indicated that combined application of high nitrogen levels and *Bradyrhizobium* further strengthened photosynthetic efficiency and drought resilience, highlighting the synergy between nutrient management and microbial inoculation in maintaining biochemical stability under water deficit^69,70^. Therefore, this study investigated the effects of nitrogen fertilizer levels and *Bradyrhizobium* inoculation on some phonological, biochemical, qualitative, and quantitative traits of guar (*Cyamopsis tetragonoloba* L.) under varying irrigation regimes in arid and semi-arid conditions.

## Materials and methods

### Plant materials

In this study, the effects of three levels of chemical nitrogen fertilizer and symbiotic bacteria on several quantitative and qualitative traits of guar (*Cyamopsis tetragonoloba* L.) under different water-deficit regimes were investigated using a split-factorial arrangement in a randomized complete block design (RCBD) with three replications. Guar seeds (RGC-1031 from India) used in this study were obtained from the University of Birjand, Iran, as institutional research material. No specific collection permit or license was required because the plant material was sourced from institutional resources rather than wild populations. Formal taxonomic identification was confirmed by Dr. Hamidreza Ramazani, faculty member at the University of Birjand. At the time of the study, a voucher specimen had not been deposited in a public herbarium and no deposition number was available.

The experiment was conducted at the research farms of the Aburaihan Campus, University of Tehran, Iran (with GPS coordinates of 35°28’29’’ N and 51°36’35’’ E) in early June 2021 and early June 2022. Experimental treatments included irrigation regimes as main plots (full irrigation, deficit irrigation up to 50% of the flowering stage, and deficit irrigation up to 50% of the podding stage), and nitrogen fertilizer levels (control, 50, and 100 kg N ha^− 1^) combined with seed inoculation using *Bradyrhizobium japonicum* strains (non-inoculation, RS-150, and RS-153) as subplots. The bacterial inoculants were liquid formulations supplied by Green Biotech Company (Qom Province, Iran), and guar seeds were soaked in the inoculant solutions for 12–24 h before sowing, following the manufacturer’s instructions. *Bradyrhizobium japonicum*, previously reported as effective symbionts for guar, were used to evaluate their potential effects on plant growth, yield, and forage quality.

Before sowing, a composite soil sample was collected from the experimental field to determine soil physicochemical properties and fertilizer requirements (Table 1). Based on soil test results, phosphorus (triple superphosphate at 50 kg ha^−1^), potassium (potassium sulfate at 170 kg ha^− 1^), nitrogen (75 kg ha^− 1^ urea 46%), and sulfur (220 kg ha^−1^) were uniformly applied to all plots following primary tillage. One-third of the nitrogen fertilizer was applied at planting, with the remainder top-dressed at the beginning of rapid growth and early podding stages. The pedigree, origin, and key agronomic characteristics of the guar cultivar used are presented in Table 2.

**Table 1:** Physico-chemical properties of the studied soil.

Soil texture	pH	Ec (dS.m^− 1^)	Organic Carbon (%)	Total Nitrogen (%)	*P* (mg.kg^− 1^)	K (mg.kg^− 1^)	Cu (ppm)	Fe (ppm)	Zn (ppm)	Mn (ppm)
Loam-Clay	8.19	7.61	1.45	0.14	79	732	0.87	4.32	2.32	7.88
Fertilizer recommendations based on soil test										
		N (kg ha^− 1^)		P (kg ha^− 1^)		K (kg ha^− 1^)	S (kg ha^− 1^)			
		75		50		170	220			

**Table 2:** Pedigree and key characteristics of guar (*Cyamopsis tetragonoloba*) cultivar RGC-1031.

Genotype/cultivar	Type	Origin	Pedigree	Key characteristics/adaptation
RGC-1031 (Guar Kranti)	Cluster bean (guar)	ARS Durgapura, Rajasthan, India	RGC-936 × (RGC-986 / P-10)	Dual-purpose (grain + fodder) cultivar; moderately tall; 90–100 days to maturity; tolerant to drought and high temperature; well adapted to semi-arid, rain-fed regions

Planting rows were spaced 50 cm apart with 5 cm between plants within rows, in plots measuring 3 × 2 m, corresponding to approximately 400,000 plants ha^− 1^. Field irrigation was applied via a drip system, with full irrigation every four days and deficit irrigation every eight days, corresponding to 50% water reduction at flowering and podding stages. Weeding was also carried out manually three times during the rapid growth, flowering, and podding stages. At the end of the experiment (in both years), various phenological, qualitative, and quantitative traits were measured. Biochemical analyses were performed at specific growth stages as indicated in Table 3 (BBCH scale) to capture peak metabolic activity and optimal forage quality. Photosynthetic pigments (Chl a, Chl b, Chl T, and CAR), leaf proline content, soluble sugars, antioxidant enzyme activities (CAT and SOD), grain protein, and forage quality parameters (crude protein, ADF, NDF, dry matter digestibility, and metabolizable energy) were quantified according to the BBCH stages listed in Table 3^71^.

**Table 3:** Growth stages of guar (BBCH scale) for biochemical analyses.

Parameter/trait	Growth stage (phenophase)	BBCH code	Description
Photosynthetic pigments (Chl a, Chl b, T Chl, and CARs)	60–69	Flowering	Full flower opening; leaves fully expanded; photosynthetic and metabolic activity at maximum. Carotenoids act as accessory pigments and protect against oxidative stress
Non-enzymatic antioxidants (Leaf proline content and soluble sugars)	60–69	Flowering	Leaves were fully developed; active osmotic adjustment and antioxidative response under stress conditions
Enzymatic activities (CAT and SOD)	60–69	Flowering	Peak antioxidant enzyme activity corresponds to high metabolic and oxidative demand during flowering
Grain protein	90–99	Physiological maturity	Fully mature grains with maximum accumulation of storage compounds (proteins)
Crude protein (Cr Pr)	71–75	Early to mid-milk stage (beginning of grain filling; milky endosperm)	Protein content begins to decline rapidly after BBCH 75 due to dilution by structural carbohydrates
Acid detergent fiber (ADF)	75–83	Late milk to early dough stage	ADF starts to rise sharply after BBCH 83 as lignin deposition increases
Neutral detergent fiber (NDF)	75–83	Late milk to early dough stage	NDF increases gradually, reaching moderate levels before senescence
Dry matter digestibility (DMD)	75–83	Late milk to early dough stage	DMD is highest during early grain filling and starts declining as structural tissues mature
Metabolizable energy (ME)	75–83	Late milk to early dough stage	ME peaks when digestibility is high and lignification is still moderate

###  Phenological traits and yield-related traits

Phenological traits included days to 50% flowering (D50F), days to 50% podding (D50P), and days to physiological maturity (DH). These traits were recorded in the field for each plot, based on the number of days from sowing to the corresponding stage: flowers observed in at least 50% of plants, pods observed in at least 50% of plants, and 95% of pods fully matured, respectively. Also, Grain yield and yield components (number of grains per pod, 1000-grain weight, and grain yield) were determined by harvesting all plants within a 1-m² area from the central part of each plot to avoid edge effects. Measurements were conducted at physiological maturity, and all values were expressed per hectare.

###  Qualitative and fodder-related attributes

In addition, photosynthetic pigments (chlorophyll a, chlorophyll b, and total chlorophyll), carotenoids (CAR), grain protein (GPr), soluble sugars (SS), and proline content (PC) were determined. Furthermore, to evaluate the effects of treatments on forage quality and to compare among treatments, forage samples were collected at the 50% podding stage from the full irrigation and deficit irrigation (up to 50% podding) treatments, and forage quality parameters, including crude protein (CPro), acid detergent fiber (ADF), neutral detergent fiber (NDF), ash percentage, dry matter digestibility (DMD), and metabolizable energy (ME), were analyzed. Forage quality analysis was restricted to the full irrigation and deficit irrigation (up to 50% podding) treatments, representing the best and worst water regimes. This selection was made to illustrate the most contrasting responses while keeping the experiment manageable in terms of laboratory analysis.

#### Photosynthetic pigments (Chl a, Chl b, Chl T, and CAR)

Chl a, Chl b, Chl T, and CAR traits were quantified following the procedures described by Arnon^72^ and Lichtenthaler^73^. For each sample, 0.5 g of fresh leaf blade tissue was weighed and homogenized in a mortar using 80% aqueous acetone. The homogenate was filtered through filter paper into a volumetric flask, and the residue was re-homogenized and filtered again to ensure complete pigment extraction. The combined filtrate was then adjusted to a final volume of 10 ml with 80% acetone. Pigment concentrations were determined spectrophotometrically using a Jenway 6300 spectrophotometer at 645, 663, and 470 nm. 80% aqueous acetone served as the blank. Chlorophyll a, chlorophyll b, total chlorophyll, and carotenoid contents were subsequently calculated using the standard equations provided by the cited references.

1 $$Chlorophyll\:a\:\left({mg.g}^{-1}\:FW\right)=\frac{[\left(12.7\:\times\:\:D663\right)-\left(2.69\:\times\:\:D645\right)]\:\times\:\:V}{1000\:\times\:\:W}$$

2 $$\:Chlorophyll\:b\:\left({mg.g}^{-1}\:FW\right)=\frac{[\left(22.9\:\times\:\:D645\right)-\left(4.93\:\times\:\:D663\right)]\:\times\:\:V}{1000\:\times\:\:W}$$

3 $$\:Total\:Chlorophyll\:\left({mg.g}^{-1}\:FW\right)=\frac{[\left(20.2\:\times\:\:D645\right)-\left(8.02\:\times\:\:D663\right)]\:\times\:\:V}{1000\:\times\:\:W}$$

4 $$\:Carotenoids\:\left({mg.g}^{-1}\:FW\right)=\frac{\left[\left(1000\:\times\:\:D470)-(1.82\:\times\:\:Chl.a)-(85.02\:\times\:\:Chl.b\right)\right]}{198}$$

Where V represents the final volume of the 80% acetone extract (mL), W is the fresh leaf tissue weight (g), and D is the absorbance measured at the specified wavelengths.

#### Proline content (PC)

Proline content was quantified following the method described by Bates et al.^74^. For each sample, 0.5 g of fresh leaf blade tissue was weighed and homogenized in 10 ml of 3% aqueous sulfosalicylic acid. The resulting homogenate was filtered through Whatman filter paper to obtain a clear extract. Subsequently, 2 ml of acid-ninhydrin solution and 2 ml of glacial acetic acid were added to 2 ml of the filtrate, and the mixture was heated at 100 °C for 60 min. After incubation, the tubes were immediately cooled in an ice bath for 30 min. Thereafter, 4 ml of toluene was added, and the solution was thoroughly mixed for 15–20 s. Two distinct phases were formed, and the upper toluene layer was carefully collected. Absorbance was measured at 520 nm using a spectrophotometer, and proline concentration was calculated from a standard curve prepared with 0, 50, 100, 200, and 250 µM proline. Final values were expressed as µM per gram of fresh leaf tissue.

5 $$\:Proline\:content\:({\upmu\:}\mathrm{M}.{g}^{-1}FW)=\frac{\left[\frac{\left({{\upmu\:}\mathrm{g}.\mathrm{m}\mathrm{l}}^{-1}\:\mathrm{p}\mathrm{r}\mathrm{o}\mathrm{l}\mathrm{i}\mathrm{n}\mathrm{e}\:\times\:\:\mathrm{m}\mathrm{l}\:\mathrm{t}\mathrm{o}\mathrm{l}\mathrm{u}\mathrm{e}\mathrm{n}\mathrm{e}\right)}{115.5\:{{\upmu\:}\mathrm{g}.{\upmu\:}\mathrm{m}\mathrm{o}\mathrm{l}\mathrm{e}}^{-1}}\right]}{\left[\frac{\left(\mathrm{g}\:\mathrm{s}\mathrm{a}\mathrm{m}\mathrm{p}\mathrm{l}\mathrm{e}\right)}{5}\right]}$$

#### Soluble sugars (SS)

Soluble sugar (SS) content was determined following the method of Yemm and Willis^75^ with minor modifications. For each sample, 0.1 g of dried leaf tissue was weighed and hydrolyzed in 5 ml of 2.5 N HCl by boiling for three hours. After cooling to room temperature, the hydrolysate was neutralized with solid sodium carbonate until effervescence ceased. The resulting solution was then diluted to 100 ml with distilled water and centrifuged at 3500 rpm for 10 min. Subsequently, 0.5 ml of the supernatant was collected and further diluted to 1 ml with distilled water. Next, 4 ml of anthrone reagent was added to each tube, and the mixture was heated in a boiling water bath for eight minutes. Absorbance was measured at 630 nm using a spectrophotometer, with a glucose-free solution serving as the blank. Soluble sugar concentrations were calculated from a standard curve prepared with known concentrations of glucose (0, 50, 100, 150, 200, and 250 µg ml^− 1^), and final values were expressed as µg per gram of dry weight (µg g^− 1^ DW).

#### Enzymatic activities of catalase (CAT) and superoxide dismutase (SOD)

#####  Catalase (CAT) activity

Catalase activity was measured following the method of Bailly et al.^76^ with minor modifications. Leaf tissues (1 g) were homogenized in 3 mL of 50 mM potassium phosphate buffer (pH 7.2) containing 1 mM EDTA, 1 mM PMSF, and 1% PVP. The homogenate was centrifuged at 14,000 × g for 15 min at 4 °C, and the supernatant was used for the assay. The reaction mixture contained 25 mM potassium phosphate buffer (pH 7.0), 10 mM H₂O₂, and enzyme extract. Catalase activity was determined based on the rate of H₂O₂ decomposition at 240 nm using an extinction coefficient of ε = 0.036 Mm^− 1^ cm^− 1^. One unit of CAT activity was defined as the amount of enzyme that decomposes 1 µmol of H₂O₂ per minute per mg of protein.

##### Superoxide dismutase (SOD) activity

SOD activity was assayed according to Giannopolitis and Ries^77^ with minor modifications. At first, fresh leaf tissue (1 g) was homogenized in 50 mM phosphate buffer containing 0.013 mM methionine, 0.1 µM EDTA, and 2 µM riboflavin under complete darkness. After adding riboflavin, 3 mL of the reaction mixture was transferred to a test tube, and 100 µL of the enzyme extract was added. Samples were irradiated under a light source at 30 cm for 16 min, and absorbance was read at 560 nm against a blank. SOD activity was expressed as units per mg protein, with one unit defined as the amount of enzyme required to inhibit 50% of superoxide-mediated reactions.

#### Grain protein (GPr) percentage

To measure grain protein and crude protein, the amount of N was first determined in the studied samples using the Kjeldahl method and calculated using the following Eqs.^78,79^.

6 $$\:Protein\:percentage\:\left(\%\right)=\mathrm{\%}\:\mathrm{K}\mathrm{j}\mathrm{e}\mathrm{l}\mathrm{d}\mathrm{a}\mathrm{h}\mathrm{l}\:\mathrm{N}\:\times\:\:\mathrm{F}$$

Where N represents the nitrogen content of the grain or the whole plant, as determined by the Kjeldahl method, and F is the conversion factor used to calculate protein, set at 6.25 for legumes.

#### Ash percentage

The percentage of ash was measured based on the method proposed by Thiex et al.^79^ with slight modifications. Therefore, 2 g of each sample was first weighed in dried glass crucibles and burned to ash in a furnace at 550 °C for 3 h. Then, the crucibles were quickly cooled in a desiccator, and the ash percentage for each treatment was calculated as follows.

7 $$\:Ash\:\left(\%\right)=\frac{Z-X}{Y-X}\:\times\:\:100$$

In which X=weight of empty crucible; Y=weight of crucible + sample; Z=weight of crucible + ash.

#### Neutral detergent fiber (NDF), acid detergent fiber (ADF), and dry matter digestibility (DMD)

Neutral detergent fiber (NDF) and acid detergent fiber (ADF) were determined to assess forage quality following the procedures described by Van Soest^80^ with minor modifications. For NDF, samples were treated with a neutral detergent solution to remove soluble cell contents, leaving the cell wall components (cellulose, hemicellulose, and lignin) as residue, which was then weighed and expressed as a percentage of dry matter. ADF was measured by treating samples with an acid detergent solution, which removes hemicellulose and soluble components, leaving cellulose and lignin as residue; the remaining material was then weighed and expressed as a percentage of dry matter. Dry matter digestibility (DMD) was subsequently estimated using the relationship between ADF and N% according to Oddy et al.^81^.

8 $$\:DMD\:\left(\%\right)=83.58-0.824\:ADF\%+2.262\:N\%$$

#### Metabolizable energy (ME)

Metabolizable energy (ME) was calculated using the equations provided by the Standing Committee on Agriculture^82^ and Williams et al.^83^ as follows (Eq. 8):

9 $$\:ME\:\left(MJ.{kg}^{-1}\right)=0.17DMD\%-2$$

### Quantitative traits (yield and yield components)

In addition, grain numbers (GN), 1000-grain weight (TGW), and grain yield (GY) were calculated based on crop yields per m^2^ at the physiological maturity stage. Yield and its components were evaluated based on the plants of each plot (according to the harvest area) after determining edge effects.

### Statistical analysis

The data were analyzed using SAS software (version 9.4), and treatment means were compared using the LSD test at *p* ≤ 0.05. Homogeneity of variances between both years was assessed using Bartlett’s test prior to analysis. Since significant heterogeneity of variances was detected, the assumption of homogeneity required for combined analysis was not satisfied; therefore, the data were analyzed separately for each experimental year. Graphs were also prepared using Microsoft Excel (2013).

## Results

### Phonological traits (days to 50% flowering, days to 50% podding, and days to harvesting)

The ANOVA results for phenological traits (D50F, D50P, and DH) across the two experimental years are presented in Table 4. Irrigation regime (IR) significantly affected all traits in both years (*p < 0.01*). In addition, nitrogen fertilizer (NF) significantly influenced D50F in both years (*p < 0.05*), whereas its effects on D50P and DH were significant only in the first year. In contrast, *Bradyrhizobium* inoculation and all interaction effects were not significant for any of the studied traits.

**Table 4:** Effects of IR, NF levels, and BRh inoculation conditions on days to 50% flowering, days to 50% podding, and days to harvesting of guar.

Source of variation	df	D50F		D50P		DH	
		First year	Second year	First year	Second year	First year	Second year
Block	2	29.05 ns	4.61 ns	20.04 ns	3.79 ns	7.7 ns	2.04 ns
Irrigation regimes (IR)	2	109.12*	487.46**	147.82**	759.46**	551.44**	988.59**
*Ea*	4	17.29	14.14	30.41	5.86	13.59	12.07
Nitrogen fertilizer (NF) levels	2	112.75*	110.42*	122.11*	53.09 ns	180.26*	203.26 ns
*Bradyrhizobium* Inoculation (BRh) conditions	2	7.46 ns	7.75 ns	11.15 ns	2.68 ns	40.11 ns	30.26 ns
Irrigation × NF levels (IR × NF)	4	3.94 ns	4.24 ns	10.93 ns	7.94 ns	6.2 ns	32.74 ns
IR × BRh	4	0.20 ns	0.22 ns	2.35 ns	3.59 ns	1.17 ns	3.24 ns
NF × BRh	4	0.61 ns	0.51 ns	2.37 ns	2.61 ns	8.15 ns	10.19 ns
IR × NF × BRh	8	0.82 ns	0.88 ns	2.02 ns	1.79 ns	2.4 ns	7.92 ns
*Eb*	48	23.32	27.92	24.26	30.7	55.88	78.12
Coefficient of Variation (CV %)		9.43	9.79	7.94	8.15	7.19	8.25

ns: non-significant; * and **: significant at *p* < 0.05 and *p* < 0.01, respectively.

**Fig. 1:**
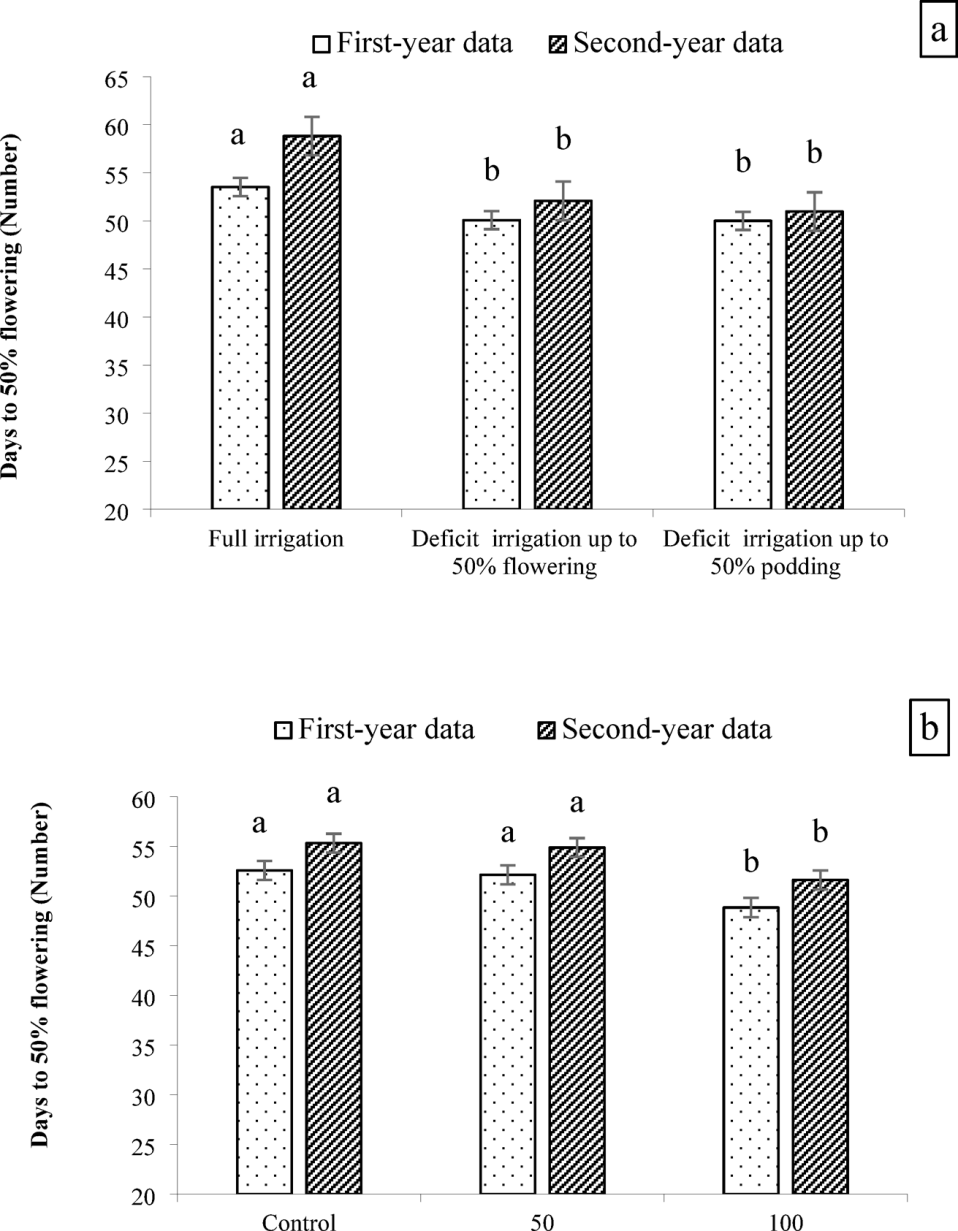
Effects of (**a**) irrigation regimes and (**b**) nitrogen fertilizer levels on days to 50% flowering (D50F) in the guar plant. Means followed by the same letter within each column do not differ significantly at *p* < 0.05.

Mean comparisons (Fig. 1a) showed that full irrigation regime consistently resulted in the longest D50F in both years, with 53.52 days in the first year and 58.82 days in the second year, which were significantly longer than the corresponding deficit irrigation treatments of 50.07 and 50 days in the first year and 52.07 and 50.96 days in the second year of the experiment, respectively. In addition, increasing nitrogen levels significantly reduced D50F (Fig. 1b). Results indicated that the application of 100 kg N ha^− 1^ produced the shortest flowering period with 48.85 days in the first year of the experiment and 51.63 days in the second year compared with 52.59 and 55.33 days for the control and 52.15 and 54.89 days for the 50 kg N ha^− 1^ treatment, respectively.

**Fig. 2:**
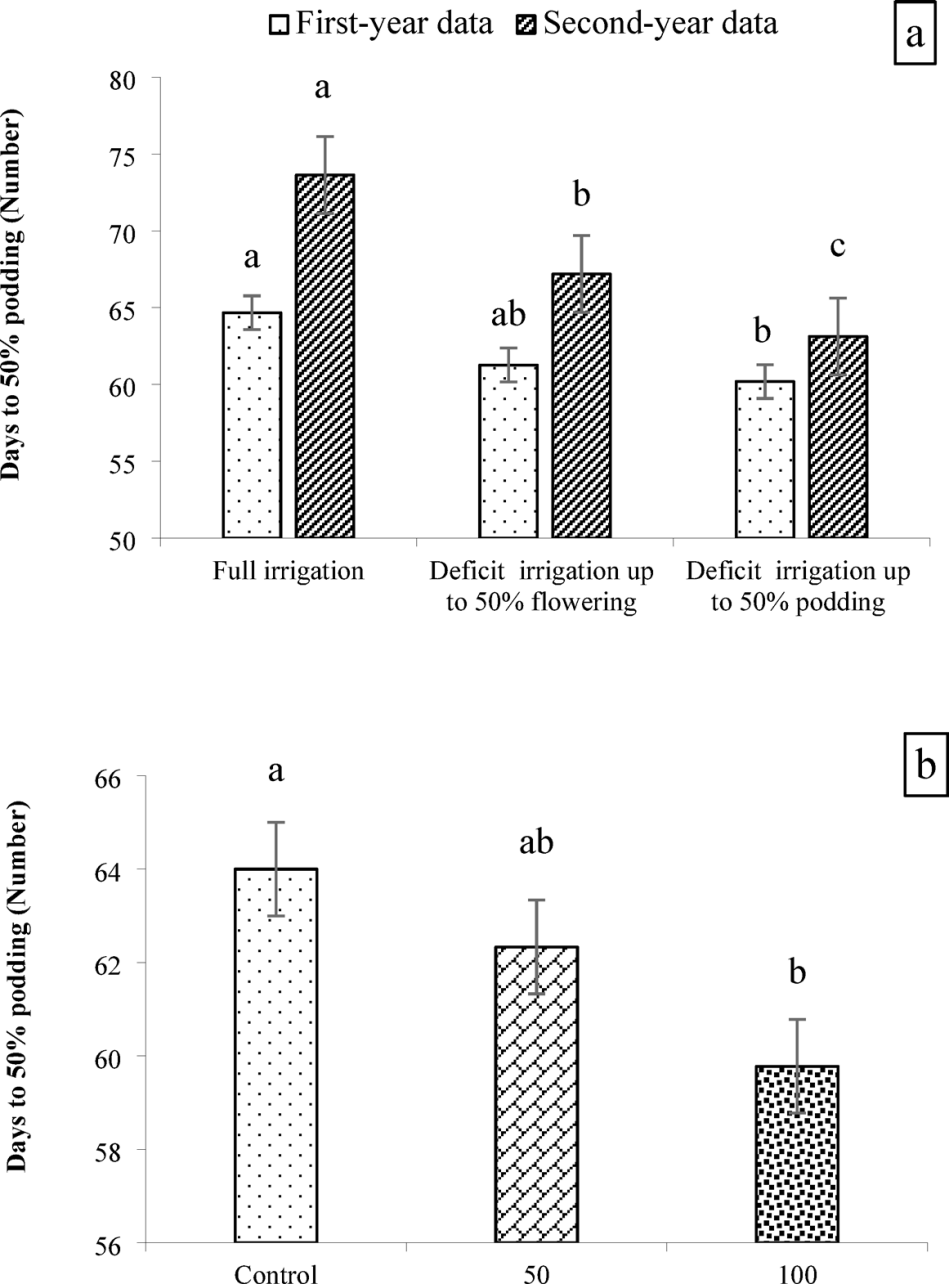
Effects of (**a**) irrigation regimes and (**b**) nitrogen fertilizer levels on days to 50% podding (D50P) in the guar plant. Means followed by the same letter within each column do not differ significantly at *p* < 0.05.

Considering the significant effects of IR on D50P (Table 4), deficit irrigation accelerated podding compared to full IR. The shortest D50P was observed under deficit IR up to 50% podding (60.19 and 63.11 days according to the first and second-year data, respectively), whereas full IR resulted in the longest D50P (64.67 and 73.63 days; Fig. 2a). In addition, the application of NF significantly affected D50P only in the first year, where 100 kg N ha^− 1^ shortened the podding period to 59.78 days compared to 64 days in the control and 61.43 days at 50 kg N ha^− 1^ (reductions of 7.06% and 2.68%), indicating that NF accelerated podding onset under these conditions. No significant NF effects were observed in the second year (Fig. 2b).

**Fig. 3:**
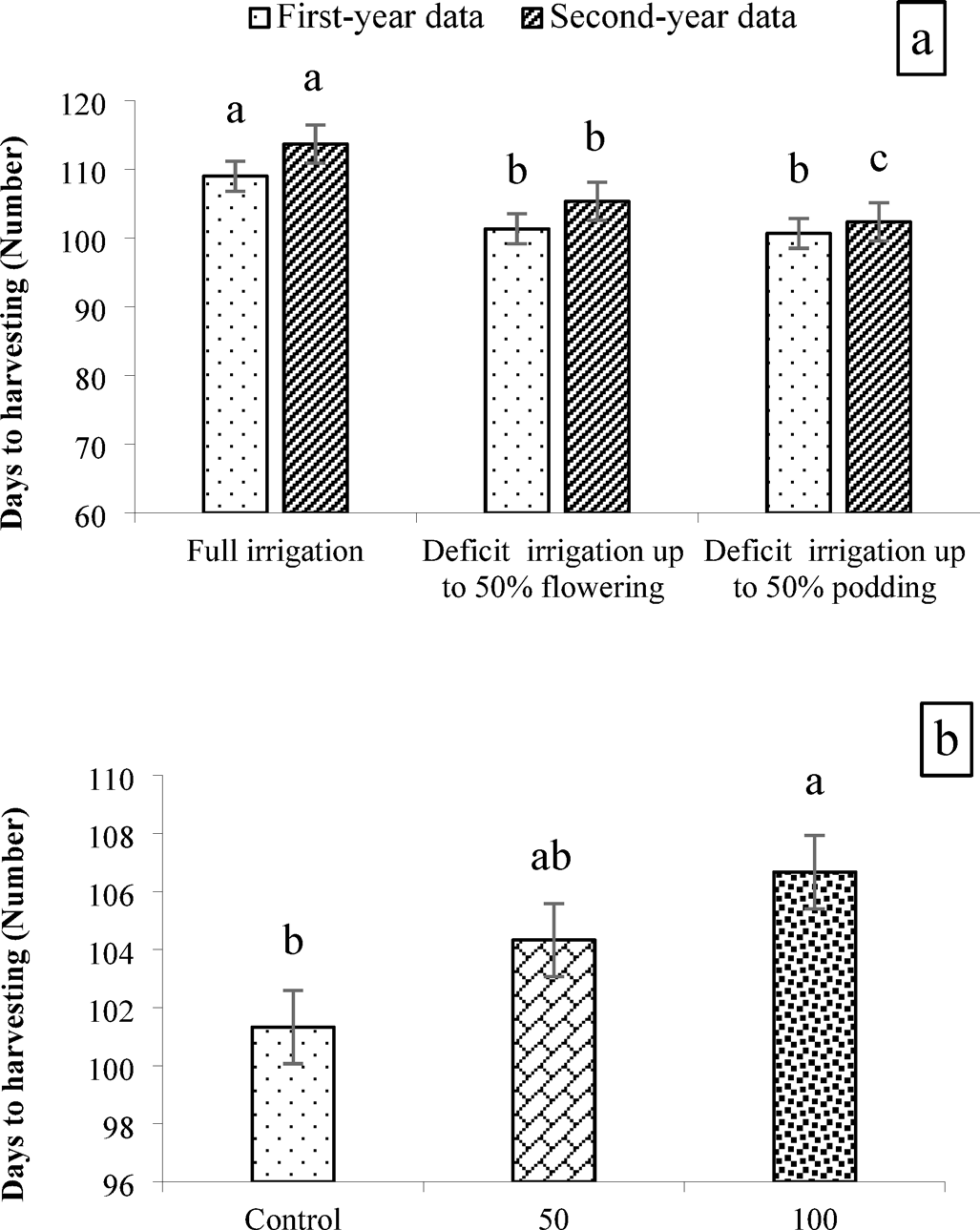
Effects of (**a**) irrigation regimes and (**b**) nitrogen fertilizer levels on days to harvesting (physiological maturity; DH) in the guar plant. Means followed by the same letter within each column do not differ significantly at *p* < 0.05.

ANOVA results (Table 4) indicated that IR significantly affected DH in both years, while NF influenced DH only in the first year. DH was consistently longer under full IR (109 days in the first year and 113.67 days in the second year) than under deficit IR, which shortened the growth period (101.33 days for deficit IR up to 50% flowering and 100.67 days for deficit IR up to 50% podding in the first year; 105.33 and 102.33 days, respectively, in the second year; Fig. 3a), reflecting accelerated development under water deficit as a drought-escape strategy. In the first year, NF application slightly prolonged DH, with 100 kg N ha^− 1^ resulting in 106.67 days, compared to 101.33 days in the control and 104.33 days at 50 kg N ha^− 1^ (Fig. 3b), indicating that adequate nitrogen extended vegetative and reproductive growth. This effect likely reflects the indeterminate growth habit of guar and the role of nitrogen in delaying leaf senescence and maintaining photosynthetic activity, allowing plants to sustain growth before reaching physiological maturity.

### Biochemical indices

####  Photosynthetic pigments (Chl a, Chl b, Chl T, and CAR)

Analysis of variance of the treatments on biochemical indices (Table 5) showed that Chl a content was significantly affected by IR, NF, and BRh inoculation in both years (*p < 0.01*), with a significant NF × BRh interaction in the first year (*p < 0.05*). In addition, Chl b was influenced by IR, NF, and IR × NF, IR × BRh interactions (*p < 0.01*), as well as NF × BRh in the first year of the experiment (*p < 0.05*). According to the second-year data, IR, NF, and the interactions IR × BRh and NF × BRh were significant (*p < 0.01*) on the Chl b content. Also, Chl T was strongly affected by IR, NF, BRh inoculation, and the IR × BRh interaction in both years, with NF × BRh significant in the first year (*p < 0.01*). Furthermore, the CAR content was significantly influenced by IR and NF in both years (*p < 0.01*) and by BRh inoculation (*p < 0.05*), whereas all investigated interactions had no significant effects on CAR levels in either year (Table 5).

**Table 5:** Effects of irrigation regimes, NF levels, and inoculation/non-inoculation conditions on photosynthetic pigments.

Source of variation	df	Chl a		Chl b		Chl T		CAR	
		First year	Second year	First year	Second year	First year	Second year	First year	Second year
Block	2	0.82ns	1.61ns	0.16*	0.24*	0.26ns	0.96ns	1.64ns	0.16ns
Irrigation regimes (IR)	2	9.4**	114.89**	0.62**	0.67**	5.82**	98.45**	17.5**	16.51**
Ea	4	2.16	2.26	0.04	0.06	1.76	1.72	0.88	0.56
NF levels	2	36.98**	84.47**	3.36**	4.8**	61.09**	128.4**	15.3**	16.08**
Inoculation conditions	2	21.87**	31.93**	0.06ns	0.07ns	21.05**	31.96**	2.72*	3.12*
IR × NF	4	0.11ns	0.66ns	0.33**	0.1ns	0.33ns	0.32ns	0.1ns	0.81ns
IR × BRh	4	1.05ns	3.55*	0.74**	0.64**	2.29**	3.58**	0.13ns	0.25ns
NF × BRh	4	1.88*	1.74ns	0.14*	0.22**	2.46**	2.19ns	0.25ns	0.31ns
IR × NF × BRh	8	0.08ns	0.91ns	0.05ns	0.09ns	0.17ns	1.46ns	0.39ns	0.17ns
Eb	48	0.72	1.02	0.03	0.05	0.61	0.9	0.65	0.61
CV (%)		12.71	16.37	9.54	13.82	9.16	12.06	17.3	20.28

ns: non-significant; * and **: significant at *p* < 0.05 and *p* < 0.01, respectively.

**Fig. 4:**
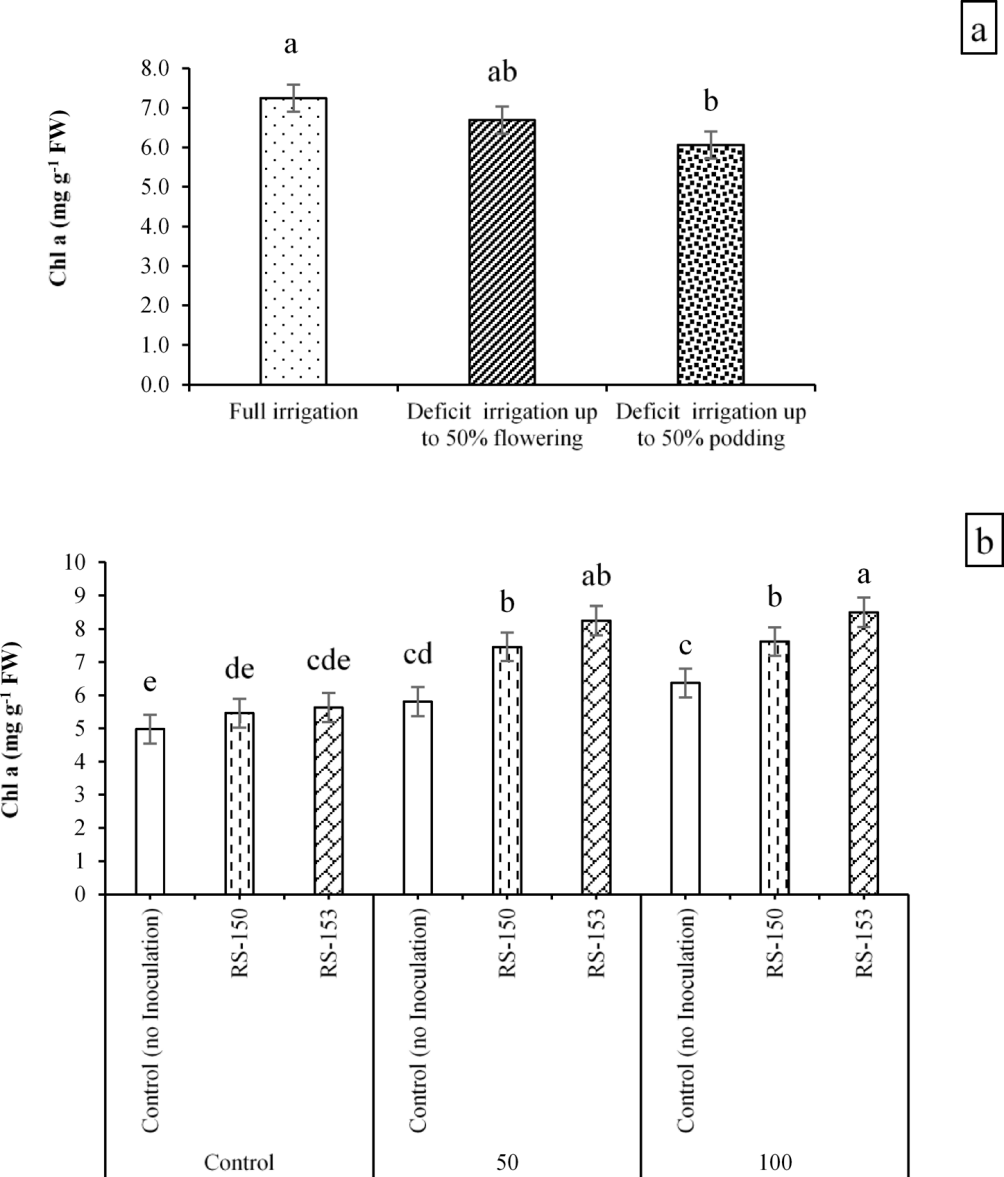
Effects of (**a**) IR and (**b**) NF × BRh interaction on the content of Chl a. (adapted from first-year data). Means followed by the same letter within each column do not differ significantly at *p* < 0.05.

The first-year mean comparisons for Chl a content under different irrigation regimes (Fig. 4a) indicated that drought stress significantly reduced Chl a. The highest and lowest values (7.24 and 6.06 mg g^− 1^ FW) were observed under full irrigation and deficit irrigation up to 50% podding, respectively. No significant difference was found between the two deficit irrigation treatments (6.69 mg g^− 1^ FW for deficit irrigation up to 50% flowering and 6.06 mg g^− 1^ FW for up to 50% podding). Overall, deficit irrigation up to 50% podding reduced Chl a content by 16.30% relative to full irrigation and by 9.42% relative to deficit irrigation up to 50% flowering. Regarding the NF × BRh interaction (first-year data; Fig. 4b), the highest Chl a values were recorded for 100 kg N N ha^− 1^ × RS-153 (8.50 mg g^− 1^ FW) and 50 kg N N ha^− 1^ × RS-153 (8.25 mg g^− 1^ FW) interactions. In contrast, the lowest value (4.89 mg g^− 1^ FW) was observed under the treatment without N fertilizer × no seed inoculation.

**Fig. 5:**
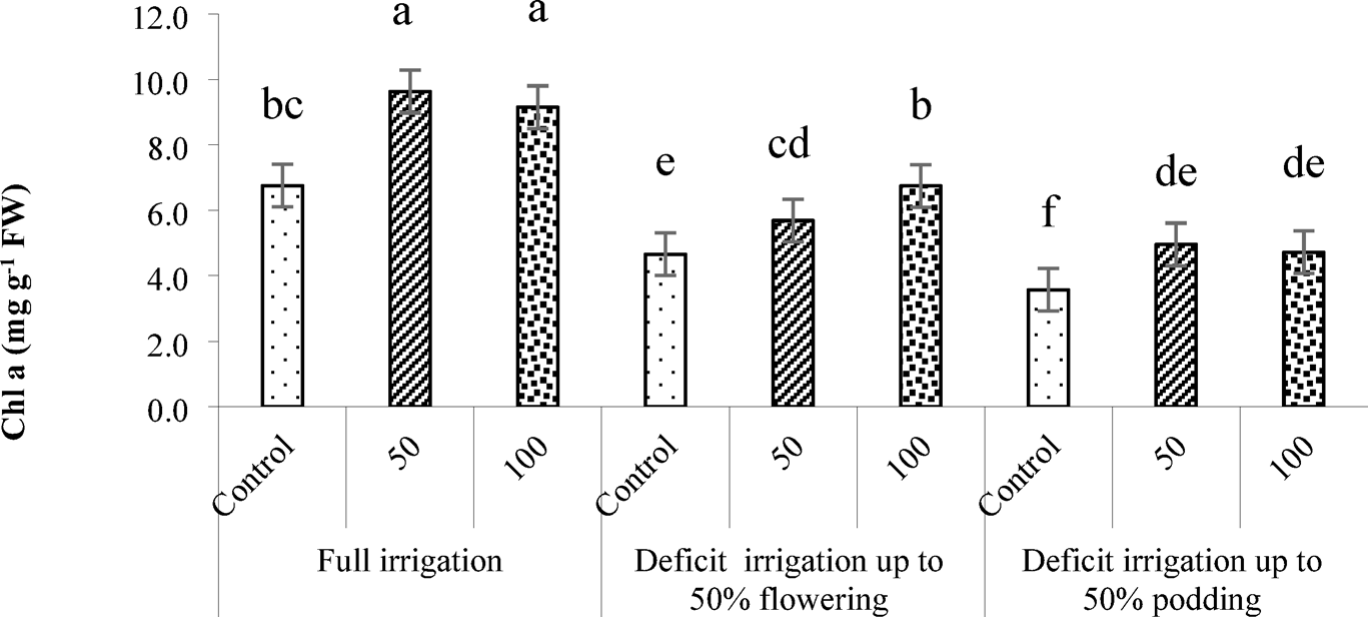
Effects of the IR × NF interaction on Chl a (adapted from second-year data). Means followed by the same letter within each column do not differ significantly at *p* < 0.05.

Given the significance effects of IR × BRh interaction on the Chl a content in the second year of the research, compare means (Fig. 5) indicated that the highest values for Chl a (9.63 and 9.15 mg g^− 1^ FW), which had significant increases compared to the other interactions, were obtained for the interactions of full irrigation × seed inoculation with the RS-150 strain and full irrigation × seed inoculation with the RS-153 strain, respectively. In contrast, the significant lowest Chl a value, Chl a, which was significantly reduced compared to the others, was recorded for the interaction of deficit irrigation up to 50% podding × non-inoculation conditions with the equivalent of 3.56 mg g^− 1^ FW (Fig. 5).

**Fig. 6:**
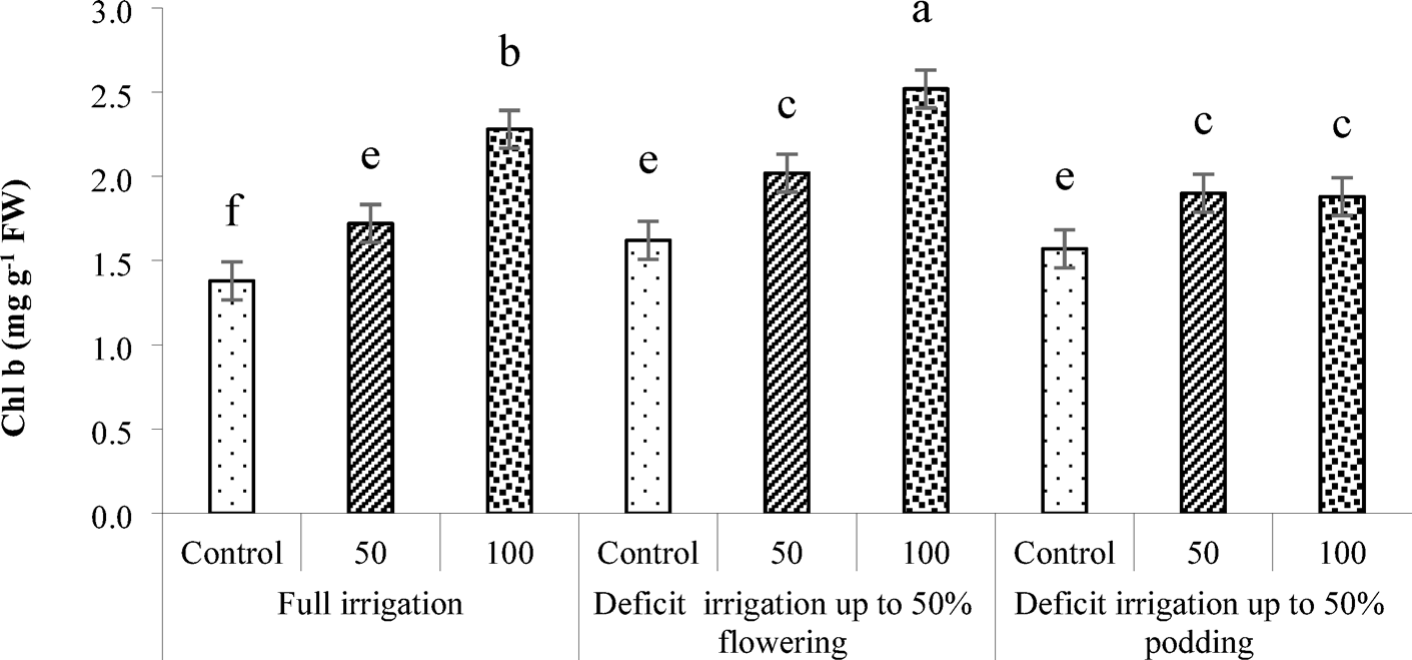
Effects of the interaction of IR × NF on the content of Chl b. Means followed by the same letter within each column do not differ significantly at *p* < 0.05.

Considering the significant effects of IR × NF, IR × BRh, and NF × BRh interactions on the content of Chl b (adopted from the first-year data; Table 5), mean squares of the IR × NF interaction (Fig. 5) revealed that the highest Chl b content (2.52 mg g^− 1^ FW) was recorded under deficit irrigation up to 50% flowering combined with 100 kg N ha^− 1^, showing a significant increase compared with other treatments. In contrast, the lowest Chl b content (1.38 mg g^− 1^ FW) was observed under full irrigation × no application of chemical N fertilizer (Fig. 6). This pattern suggests that moderate water deficit combined with adequate nitrogen supply might have stimulated Chl biosynthesis and improved N assimilation efficiency, possibly due to enhanced photosynthetic adaptation and delayed leaf senescence in guar.

At same time, mean comparisons related to the IR × BRh interaction obtained from the first year of the experiment (Table 6) showed that the highest Chl b content (2.19 mg g^− 1^ FW) was recorded for the deficit irrigation up to 50% podding × RS-150 interaction, with no significant difference from deficit irrigation up to 50% podding × RS-153 (2.09 mg g^− 1^ FW). In contrast, the lowest Chl b value (1.42 mg g^− 1^ FW) was observed under deficit irrigation up to 50% flowering × RS-153.

**Table 6:** Effects of IR × BRh interaction on the contents of Chl a, Chl b, and Chl T.

Source of variation		Chl b (mg g^− 1^ FW)		Chl T (mg g^− 1^ FW)	
		First year	Second year	First year	Second year
Irrigation regimes (IR)	Inoculation conditions				
Full irrigation	Non-Inoculation (Control)	1.66 cd	1.64 bcd	7.51 ef	8.21 b
	RS-150	1.72 ef	1.46 cde	9.47 ab	11.09 a
	RS-153	2.02 bcd	1.67 bc	10.13 a	10.81 a
Deficit irrigation up to 50% flowering	Non-Inoculation (Control)	2.06 abc	1.42 de	8.01 de	6.09 d
	RS-150	1.88 de	1.71 b	8.67 cd	7.39 bc
	RS-153	1.42 g	1.98 a	8.74 bcd	8.67 b
Deficit irrigation up to 50% podding	Non-Inoculation (Control)	1.88 f	1.35 e	7.14 f	4.91 e
	RS-150	2.19 a	2 a	8.18 de	6.95 cd
	RS-153	2.09 ab	1.85 ab	9.03 bc	6.56 cd
LSD		0.17	0.23	0.79	0.93

Means followed by the same letter within each column do not differ significantly at *p* < 0.05.

Regarding the NF × BRh interaction effects (Table 7; first-year data), the highest Chl b value (2.32 mg g^− 1^ FW) was observed under 100 kg N ha^− 1 ×^ RS-150. The lowest Chl b content (1.39 mg g^− 1^ FW) occurred under control (no N fertilizer) × RS-153, which was not significantly different from control × RS-150 (1.55 mg g^− 1^ FW). Moreover, the second-year data revealed a similar pattern, where the highest and lowest Chl b values were obtained under deficit irrigation up to 50% podding × RS-150 (2.00 mg g^− 1^ FW) and deficit irrigation up to 50% podding × non-inoculated treatment (1.35 mg g^− 1^ FW), respectively. Similar trends were also observed for Chl T (Table 7), as discussed in the following section.

**Table 7:** Effects of NF × BRh interaction on the contents of Chl a, Chl b, and Chl T (The first year of data).

Source of variation		Chl b (mg g^− 1^ FW)		Chl T (mg g^− 1^ FW)	SS (µg g^− 1^ DW)
		First year	Second year	First year	First year
NF levels (Kg ha^− 1^)	Inoculation conditions				
Control	Non-Inoculation (Control)	1.55 ef	1.33 f	6.44 e	163.65 e
	RS-150	1.63 e	1.36 f	7.09 de	160.59 e
	RS-153	1.39 f	1.1 g	7.02 de	164.75 e
50	Non-Inoculation (Control)	1.81 d	1.55 ef	7.62 d	179.23 d
	RS-150	1.83 d	1.61 de	9.29 bc	200.38 c
	RS-153	2.01 c	1.81 cd	10.25 a	210.84 ab
100	Non-Inoculation (Control)	2.24 ab	1.96 bc	8.61 c	185.29 d
	RS-150	2.32 a	2.21 a	9.94 ab	207.67 bc
	RS-153	2.12 bc	2.15 ab	10.62 a	219.07 a
LSD		0.17	0.23	0.79	10.26

Means followed by the same letter within each column do not differ significantly at *p* < 0.05.

Mean comparisons related to the IR × BRh interaction on Chl T showed that the highest and lowest values (10.13 and 7.14 mg g^− 1^ FW) were recorded for the interaction of full irrigation × RS-153 and deficit irrigation up to 50% podding × non-inoculation, respectively (Table 6). Similarly, according to the NF × BRh interaction (Table 7), the maximum and minimum Chl T (10.62 and 6.44 mg g^− 1^ FW) were obtained under 100 kg N ha^− 1^ × RS-153 and no application of NF (control) × non-inoculation conditions (control), respectively. According to the second-year data, the interaction of IR × BRh also showed a significant effect on Chl T. The highest Chl T value (11.09 mg g^− 1^ FW) was observed under full irrigation × seed inoculation with RS-150, which was not significantly different from full irrigation × seed inoculation with RS-153. Conversely, the lowest Chl T content (4.91 mg g^− 1^ FW) was recorded under deficit irrigation up to 50% podding × non-inoculation (Table 6).

For CAR contents, the first-year data (Table 8) indicated that full irrigation resulted in the highest carotenoid concentration (5.37 mg g^− 1^ FW), which was not significantly different from deficit irrigation up to 50% flowering (4.78 mg g^− 1^ FW), while the lowest CAR content (3.77 mg g^− 1^ FW) occurred under deficit irrigation up to 50% podding. Nitrogen fertilization increased CAR, with 100 kg N ha^− 1^ producing 4.98 mg g^− 1^ FW compared to 50 kg N ha^− 1^ (4.28 mg g^− 1^ FW) and the control (3.78 mg g^− 1^ FW). Seed inoculation with RS-153 and RS-150 also significantly enhanced CAR content (4.85 and 4.80 mg g^− 1^ FW, respectively) relative to non-inoculated plants. Although CAR content decreased slightly in the second year, the trend among treatments remained consistent: full irrigation (4.66 mg g^− 1^ FW) increased CAR content by 24.6% and 50.3% relative to deficit irrigation up to 50% flowering and deficit irrigation up to 50% podding, respectively, while 100 kg N ha^− 1^ enhanced CAR by 50.5% and 11.9% compared with the control and 50 kg N ha^− 1^ treatments, respectively. Seed inoculation with RS-150 also resulted in significant and non-significant increases of 19.5% and 7.5% compared with non-inoculated plants and those inoculated with RS-153, respectively (Table 8; second-year data).

**Table 8:** Effects of IR, NF levels, and BRh inoculation conditions on CAR, PC, and GPr traits.

Source of variation	CAR (mg g^− 1^ FW)		PC (µg g^− 1^ FW)	GPr (%)		CAT (µmol H_2_O_2_ mg^− 1^ protein min^− 1^)		SOD (mg protein min^− 1^)	
	First year	Second year	First year	First year	Second year	First year	Second year	First year	Second year
Irrigation regimes (IR)									
Full irrigation	5.37 a	4.66 a	4.4 c	29.27 a	26.92 a	23.98 c	31.52 c	45.27 c	56.17 c
Deficit irrigation up to 50% flowering	4.78 a	3.74 b	4.97 b	26.16 b	26.60 a	31.33 b	43.82 b	54.23 b	73.98 b
Deficit irrigation up to 50% podding	3.77 b	3.10 c	5.66 a	23.96 c	24.27 b	35.70 a	52.20 a	61.11 a	90.22a
LSD	0.71	0.57	0.44	0.59	1.27	1.66	3.14	4.07	4.59
NF levels (kg ha^− 1^)									
Control	3.78 b	2.99 c	3.49 b	24.19 c	24.29 b	34.81 a	50.90 a	56.37 a	89.27 a
50	4.97 a	4.02 b	5.77 a	25.87 b	26.61 b	28.49 b	41.87 b	52.78 b	69.33 b
100	5.18 a	4.50 a	5.77 a	29.34 a	29.89 a	27.72 b	34.76 c	51.45 b	61.75 c
Inoculation conditions									
Non-inoculation (Control)	4.28 b	3.48 b	5.31 a	24.01 b	25.21 b	32.14 a	48.52 a	57.90 a	79.09 a
RS-150	4.80 a	4.16 a	4.94 b	27.24 a	27.52 a	30.69 a	42.20 b	52.70 b	69.14 b
RS-153	4.85 a	3.87 ab	4.77 b	28.15 a	28.06 a	28.18 b	36.81 c	50.01 c	72.12 b
LSD	0.43	0.42	0.27	1.58	1.14	1.83	3.53	2.49	4.21

Means followed by the same letter within each column do not differ significantly at *p* < 0.05.

CAR: Carotenoids; PC: Proline content; GPr: Grain protein; CAT: Catalase; SOD: Superoxide dismutase.

#### Soluble sugars (SS), proline content (PC), and grain protein (GPr)

In addition to the previous findings, the ANOVA results (Table 9) indicated that SS was significantly affected by IR, NF, BRh inoculation, and interaction between NF and BRh (NF × BRh) during the first year of the experiment (*p < 0.01*). In the second year, the SS content was also significantly influenced by IR, NF, both interaction of IR × NF and IR × BRh (*p < 0.01*), as well as by BRh inoculation and the three-way interaction of IR × NF × BRh (*p < 0.05*). Moreover, IR, NF, and BRh inoculation had significant effects on PC and GPr percentage in both years (*p < 0.01*). In addition, significant differences in GPr percentage were observed among IR, NF, and BRh inoculation treatments in both experimental years (*p < 0.01*).

**Table 9:** Effects of irrigation regimes, NF levels, and inoculation/non-inoculation conditions on attributes of SS, PC, GPr, and enzymatic activities of CAT and SOD.

Source of variation	df	SS		PC		GPr		CAT		SOD	
		First year	Second year	First year	Second year	First year	Second year	First year	Second year	First year	Second year
Block	2	67.22ns	85.4ns	0.5ns	0.46ns	35.19*	26.9**	5.58ns	37.89ns	14.46ns	96.59ns
Irrigation regimes (IR)	2	21055.2**	541,258**	10.74**	171.72**	192.22**	192.16**	947.35**	2920.32**	1704.44**	7823.71**
Ea	4	25.37	24.77	0.34	0.69	0.61	2.82	4.83	17.22	29.05	36.93
Nitrogen Fertilizer (NF) levels	2	12949.14**	2379.08**	46.83**	83.52**	186.12**	214.2**	408.03**	1766.96**	175.09**	5455.04**
*Bradyrhizobium* Inoculation (BRh) conditions	2	3367.97**	1077.22*	2.02**	1.23ns	128.2**	61.57**	108.24**	928.22**	433.93**	703.9**
IR × NF	4	76.31ns	15759.76**	0.28ns	13.36**	6.1ns	1.21ns	4.66 ns	27.45 ns	16.4 ns	52.36 ns
IR × BRh	4	44.08ns	1387.28**	0.33ns	3.06**	5.89ns	0.89ns	13.6 ns	27.13 ns	3.99 ns	23.98 ns
NF × BRh	4	832.44**	778.02ns	0.04ns	0.51ns	1.51ns	6.34ns	7.86 ns	40.51 ns	19.7 ns	32.15 ns
IR × NF × BRh	8	28.95ns	728.95*	0.014ns	0.09ns	1.61ns	5.19ns	0.84 ns	19.93 ns	0.29 ns	28.92 ns
Eb	48	141.08	334.61	0.25	0.42	8.29	4.36	11.14	41.54	20.66	59.21
CV (%)		6.32	6.37	9.96	7.25	10.88	7.75	11.00	15.16	8.49	10.48

ns: non-significant; * and **: significant at *p* < 0.05 and *p* < 0.01, respectively.

Ea: Error a; Eb: Erreor b; CV: Coefficient of Variation; SS: Soluble sugars; PC: Proline content; GPr: Grain protein; CAT: Catalase; SOD: Superoxide dismutase.

**Fig. 7:**
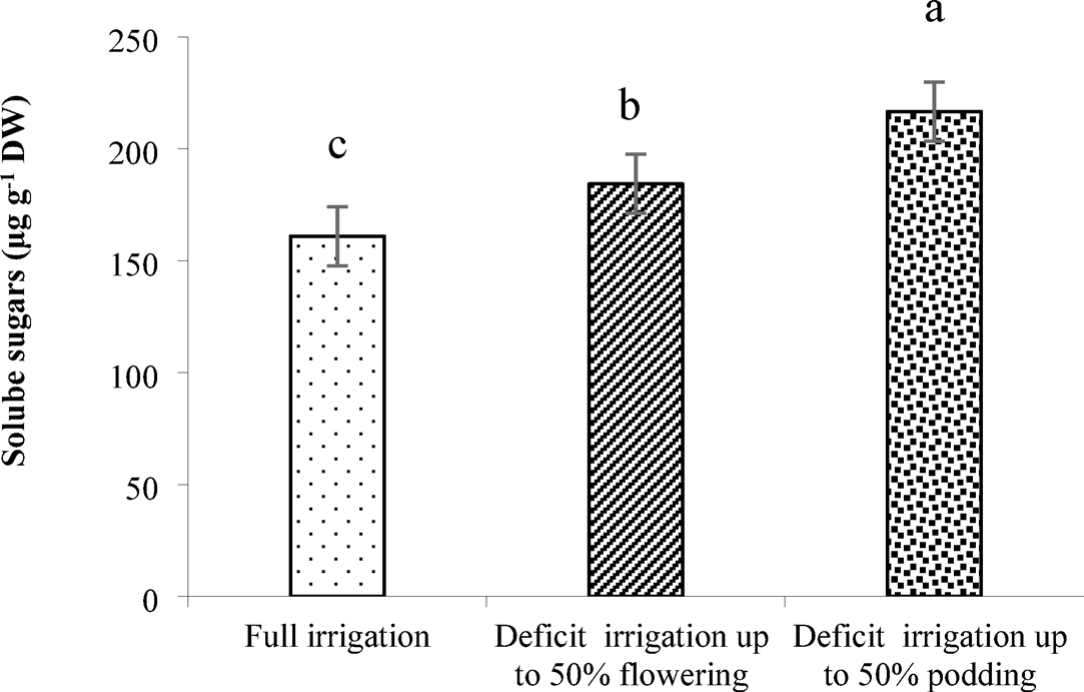
Effects of IR on the content of SS (adapted from first-year data). Means followed by the same letter within each column do not differ significantly at *p* < 0.05.

For SS content, the response of guar plants to different IR during the first year of the experiment (Fig. 7) indicated that deficit irrigation up to 50% podding produced the highest soluble sugar content (216.72 µg g^− 1^ DW), representing significant increases of 34.65% and 17.53% compared with full irrigation (160.95 µg g^− 1^ DW) and deficit irrigation up to 50% flowering (184.40 µg g^− 1^ DW), respectively. Furthermore, the NF × BRh interaction significantly influenced SS accumulation (see Table 7), with the highest SS level (219.07 µg g^− 1^ DW) recorded under 100 kg N ha^− 1^ × RS-153. In contrast, the lowest value (160.59 µg g^− 1^ DW) was observed for the control (no NF) × RS-150 interaction. These results highlight that water deficit during the podding stage strongly induces osmolyte accumulation, while the synergistic application of optimal nitrogen levels and *Bradyrhizobium* inoculation further enhances osmotic adjustment and soluble sugar accumulation in guar.

**Fig. 8:**
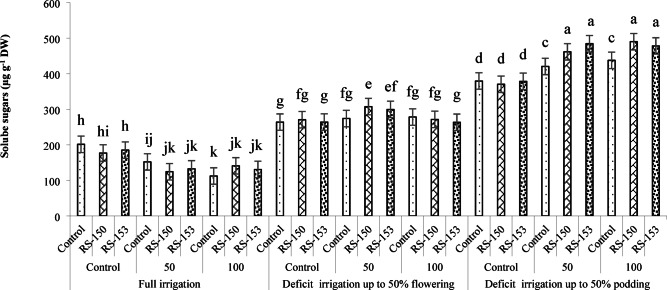
Effects of the interaction of IR × NF × BRh on the content of SS trait. Means followed by the same letter within each column do not differ significantly at *p* < 0.05.

Based on the second-year data, a significant three-way interaction of IR × NF × BRh was observed for SS (Fig. 8), showing that the highest SS content (490.16 µg g^− 1^ DW) occurred under deficit irrigation up to 50% podding × 100 kg N ha^− 1^ × RS-150, whereas the lowest value (112.41 µg g^− 1^ DW) was recorded under full irrigation × 100 kg N ha^− 1^ × non-inoculated conditions. These results indicate that the combined application of adequate nitrogen and *Rhizobium* inoculation, particularly under moderate water deficit, effectively enhances SS accumulation, likely contributing to improved osmotic adjustment and stress tolerance in guar plants.

Mean squares for the PC trait obtained from the first-year data (Table 10) showed that the highest value (5.66 µmol g^− 1^ FW) was recorded in plants subjected to deficit irrigation up to 50% podding. Regarding nitrogen fertilization, both 50 and 100 kg N ha^− 1^ treatments (averaging 5.77 µmol g^− 1^ FW) led to a 65.33% increase in leaf proline content compared with the control (3.49 µmol g^− 1^ FW). Moreover, plants under non-inoculation conditions (5.31 µmol g^− 1^ FW) exhibited significantly higher PC values compared to those inoculated with RS-150 and RS-153 strains (4.94 and 4.77 µmol g^− 1^ FW, respectively), with no significant difference between the two inoculated treatments.

**Fig. 9:**
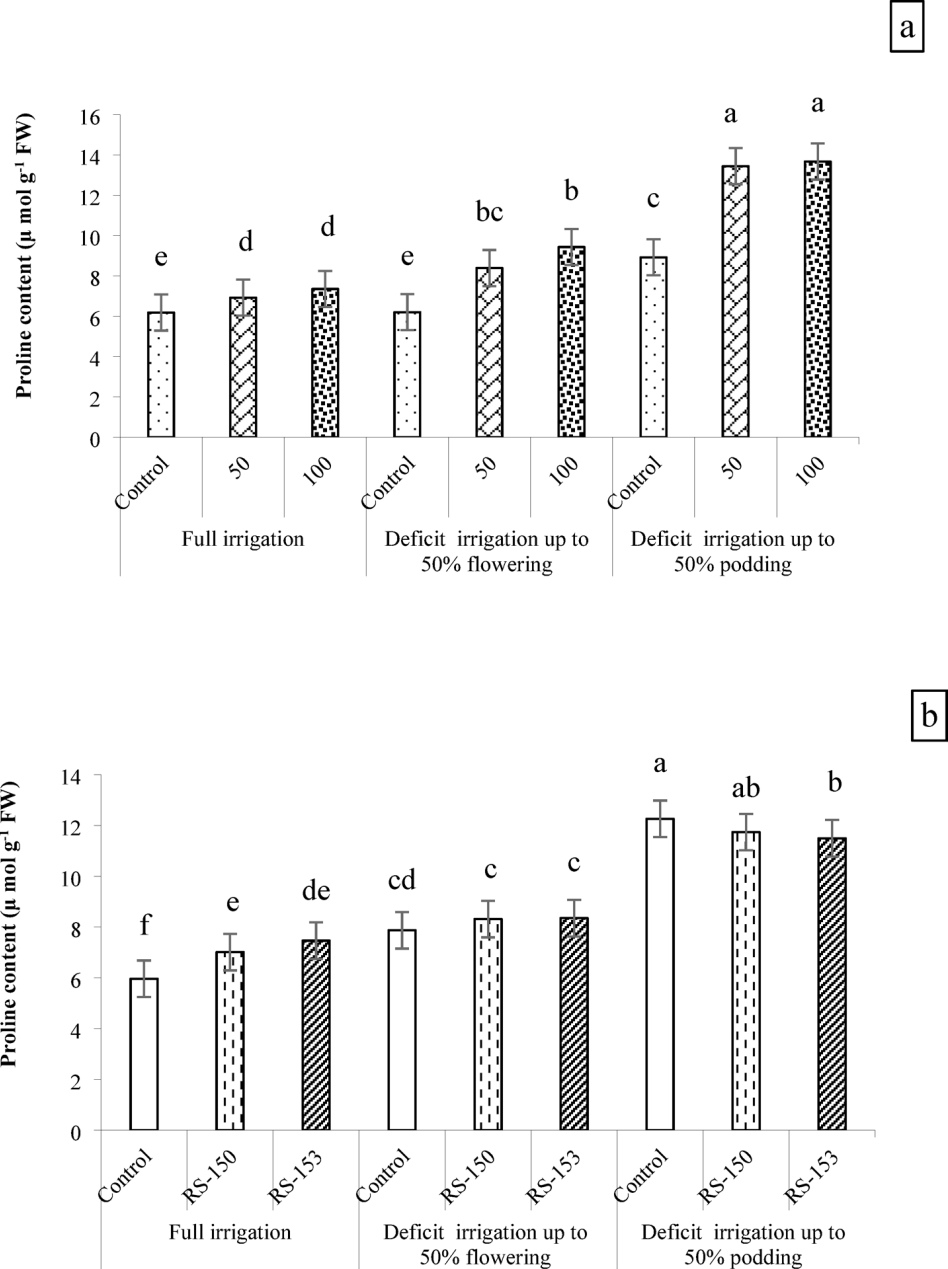
Effects of interactions of (**a**) IR × NF and (**b**) IR × BRh on the PC trait. Means followed by the same letter within each column do not differ significantly at *p* < 0.05.

In the second year of the study, PC was significantly affected by the IR × NF and IR × BRh interactions. Results of the IR × NF interaction (Fig. 9a) showed that the highest proline contents (13.67 and 13.44 µmol g^− 1^ FW) were recorded under deficit irrigation up to 50% podding combined with 100 and 50 kg N ha^− 1^, respectively. In contrast, the lowest PC (6.18 µmol g^− 1^ FW) was observed under full irrigation × non-inoculation treatment (Fig. 9a). Similarly, the IR × BRh interaction (Fig. 9b) revealed that the highest proline accumulation occurred under deficit irrigation up to 50% podding × non-inoculation, which was not significantly different from the deficit irrigation up to 50% podding × RS-150 inoculation treatment (11.74 µmol g^− 1^ FW). Conversely, the lowest significant PC (5.97 µmol g^− 1^ FW) was recorded under full irrigation × non-inoculation conditions (Fig. 9b).

According to ANOVA results, the main effects of IR, NF levels, and BRh inoculation significantly influenced GPr in both years of the experiment. In the first year (Table 8), full irrigation produced the highest GPr (29.27%), representing increases of 11.89% and 22.19% compared to deficit irrigation up to 50% flowering and 50% podding, respectively. Regarding NF application, the maximum and minimum GPr values (29.34% and 24.19%) were recorded under 100 kg N ha^− 1^ and the control (no NF), respectively. Seed inoculation with RS-153 also resulted in the highest GPr (28.15%), while non-inoculated plants showed the lowest value (24.01%). In the second year, full irrigation again resulted in the highest GPr (26.92%), which was not significantly different from deficit irrigation up to 50% flowering (26.60%), whereas deficit irrigation up to 50% podding produced the lowest GPr (24.27%). Nitrogen application exhibited a concentration-dependent effect, with 100 kg N ha^− 1^ increasing GPr by 23.06% and 12.33% compared with the control and 50 kg N ha^− 1^ treatments, respectively. Additionally, *Bradyrhizobium* inoculation significantly enhanced GPr relative to non-inoculated plants, with RS-153 (28.06%) outperforming both RS-150 (27.52%) and the non-inoculated control (25.21%).

#### Enzymatic activities of catalase (CAT) and superoxide dismutase (SOD)

The ANOVA results (Table 9) revealed that the experimental treatments had significant effects on the enzymatic activities examined in this study. Specifically, IR, NF levels, and BRh inoculation significantly influenced CAT and SOD in both years of the experiment at *p* < 0.01.

According to the first-year data (Table 8), the highest and lowest significant CAT activities (70.35 and 23.98 µmol H_2_O_2_.mg^− 1^ protein min^− 1^) and SOD activities (11.61 and 27.45 U mg^− 1^ protein min^− 1^) were observed under deficit irrigation up to 50% podding and full irrigation treatments, respectively. Regarding NF effects, increasing NF levels significantly reduced CAT and SOD activities. Under control (no NF application), CAT and SOD activities were 81 µmol H_2_O_2_.mg^− 1^ protein min^− 1^ and 37.56 U mg^− 1^ protein min^− 1^, respectively. Additionally, seed inoculation with RS-153 decreased CAT activity by 12.32% and 18.8%, and SOD activity by 13.63% and 5.10% compared with the control and RS-150 inoculation, respectively. In the second year of the experiment, mean comparisons (Table 8) showed that the highest and lowest values for CAT (20.52 and 31.52 µmol H_2_O_2_.mg^− 1^ protein min^− 1^) and SOD (21.90 and 17.56 U.mg^− 1^ protein min^− 1^) were observed under deficit irrigation up to 50% podding and full irrigation, respectively. Increasing NF levels significantly decreased CAT and SOD activities, with minimum and maximum activities observed under control (no NF application) and 100 kg N ha^− 1^, respectively. Furthermore, inoculation with *Rhizobium* strains (RS-150 and RS-153) led to a significant reduction in enzymatic activities compared to non-inoculated plants (Table 8).

### Quantitative traits of grain number (GN), 1000-grain weight (TGW), and grain yield (GY)

In general, ANOVA results (Table 10) indicated that all main factors, including IR, NF, and inoculation/non-inoculation conditions, significantly influenced TGW, GN, and GY during the first year of the study. In the second year, interactions between treatments also showed notable effects: GN was significantly affected by IR × NF (*p < 0.01*) and IR × BRh (*p < 0.05*), while GY was significantly influenced by the IR × NF interaction (*p < 0.01*).

**Table 10:** Effects of IR, NF levels, and inoculation/non-inoculation of *Rhizobium* bacteria on yield and yield components.

Sources of variations	df	GN		TGW		GY	
		First year	Second year	First year	Second year	First year	Second year
Block	2	426847.49 ns	558,479 ns	1.08 ns	34.53 ns	59422.08 ns	269631.01 ns
Irrigation regimes (IR)	2	4133915.01**	138803101.2**	159.12**	220.22**	1370373.46**	20548539.5**
Ea	4	37597.11	85619.8	6.74	17.78	16047.06	109666.86
Nitrogen Fertilizer (NF) levels	2	2414560.64**	18,270,242**	128.03**	185.46**	740600.74**	4628131.8**
*Bradyrhizobium* Inoculation (BRh) conditions	2	2197763.12**	3754212.3**	113.07**	80.79**	691384.45**	1218157.8**
IR × NF	4	57477.42 ns	3953471.6**	4.86 ns	2.12 ns	15520.94 ns	603128.49 **
IR × BRh	4	44569.29 ns	969797.5 *	0.15 ns	1.66 ns	18096.77 ns	82825.6 ns
NF × BRh	4	30671.75 ns	228938.3 ns	3.18 ns	3.68 ns	5709.68 ns	36561.09 ns
IR × NF × BRh	8	39297.75 ns	276656.8 ns	3.4 ns	2.99 ns	26836.92 ns	21894.72
Eb	48	147123.3	340506.2	11.23	12.27	28541.09	97098.02
CV (%)		9.1	9.22	11.53	12.64	13.71	16.41

ns: non-significant; * and **: significant at *p* < 0.05 and *p* < 0.01, respectively.

Based on the first-year data (Table 11), full irrigation (4662.52 grains m^− 2^) significantly increased GN m^− 2^ by 15.70% and 18.12% compared with deficit irrigation up to 50% flowering (4029.78 grains m^− 2^) and 50% podding (3947.33 grains m^− 2^), respectively. Similarly, the application of 100 kg N ha^− 1^ resulted in 4544.8 grains m^− 2^, representing significant increases of 14.65% and 10.02% relative to the control (3964 grains m⁻²) and 50 kg N ha^−1^ (4130.8 grains m⁻²), respectively. Seed inoculation with RS-150 and RS-153 strains also improved GN m^− 2^ compared with the non-inoculated control, with the highest (4467.1 grains m^− 2^) and lowest (3904.4 grains m^− 2^) values recorded for RS-153 and non-inoculated treatments, respectively. Overall, RS-153 inoculation increased GN m^− 2^ by 14.41% and 4.66% compared with non-inoculated and RS-150-inoculated plants, respectively. Regarding TGW, the highest value in the first year (31.6 g) was obtained under full irrigation, corresponding to significant increases of 9.57% and 18.07% relative to deficit irrigation up to 50% flowering and 50% podding, respectively. Application of 100 kg N ha^− 1^ increased TGW by 15.90% and 4.25% compared with the control and 50 kg N ha^−1^ treatments, respectively. Among inoculation treatments, TGW was highest in RS-153-inoculated plants (30.64 g) and lowest in the non-inoculated control (26.75 g). In the first year of the experiment, GY of guar plants ranged from 1042.1 to 1481.3 kg ha^− 1^, with the highest value recorded under full irrigation and the lowest under deficit irrigation up to 50% podding. Nitrogen fertilization also significantly influenced GY, where 100 kg N ha^− 1^ resulted in the maximum yield (1481.3 kg ha^− 1^). Similarly, inoculation treatments significantly affected GY, with the highest and lowest values observed in RS-153-inoculated (1363.60 kg ha^−1^) and non-inoculated control plants (1054.27 kg ha^− 1^), respectively (Table 11).

**Table 11:** Effects of IR, NF levels, and inoculation conditions on some traits of guar.

Sources of variations	GN (number)	TGW (g)		GY (kg ha^− 1^)
	First year	First year	Second year	First year
Irrigation regimes (IR)				
Full irrigation	4662.52 a	31.6 a	32.1 a	1481.3 a
Deficit irrigation up to 50% flowering	4029.78 b	28.84 b	28.37 b	1174.6 b
Deficit irrigation up to 50% podding	3947.33 b	26.76 c	26.49 b	1042.1 c
LSD	146.52	1.96	3.19	95.7
NF levels (kg ha^− 1^)				
Control	3964 b	26.66 b	26.4 c	1068.6 c
50	4130.8 b	29.64 b	28.93 b	1299.51 b
100	4544.8 a	30.9 a	31.64 a	1399.79 a
BRh Inoculation conditions				
Control (non-inoculation)	3904.4 b	26.75 b	27 b	1054.27 b
RS 150	4268.1 a	29.8 a	29.76 a	1280.03 a
RS 153	4467.1 a	30.64 a	30.19 a	1363.60 a
LSD	209.9	1.83	1.95	92.45

Means followed by the same letter within each column do not differ significantly at *p* < 0.05.

**Fig. 10:**
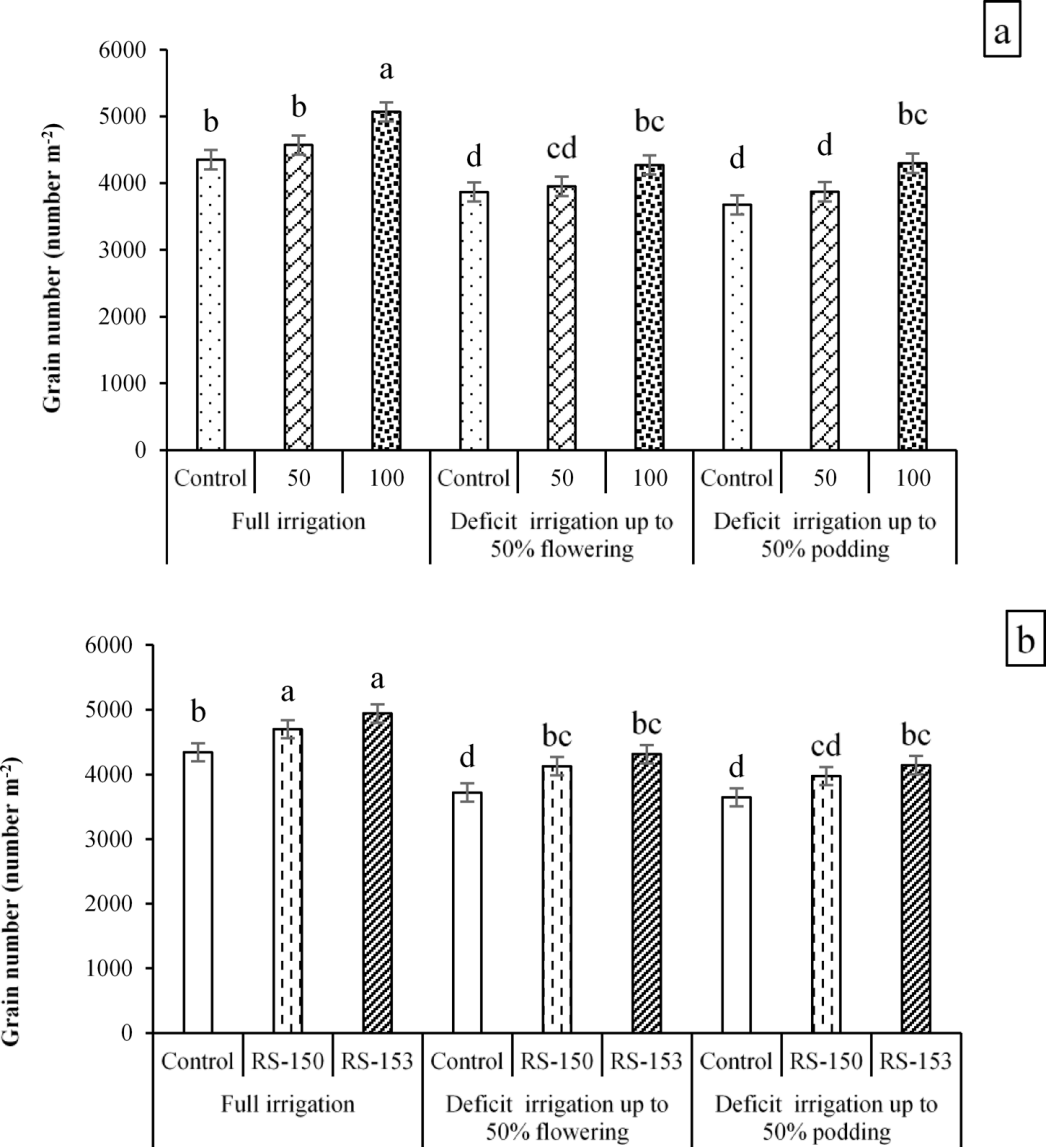
Interaction effects of (**a**) IR × NF levels and (**b**) IR × Rh inoculation conditions on GN per square meter (adapted from the second-year data). Means followed by the same letter within each column do not differ significantly at *p* < 0.05.

Based on the second-year data (Fig. 10a), GN per m² was significantly affected by the interaction of IR × NF. The highest GN (5066.33 grains) was recorded under full irrigation × 100 kg N ha^− 1^, which showed a significant increase compared to other treatment combinations. In contrast, the lowest GN (3675 grains) occurred under deficit irrigation up to 50% podding × no N application, although it was not significantly different from the combinations of deficit irrigation up to 50% flowering × no N fertilizer (3866 grains), deficit irrigation up to 50% flowering × 50 kg N ha^− 1^ (3875 grains), and deficit irrigation up to 50% podding × 50 kg N ha^− 1^ (3870.33 grains). Furthermore, according to the results of the GN trait in the second year (Fig. 10b), the highest GN (4943 grains) was obtained under full irrigation × seed inoculation with the RS-153 strain, which was statistically similar to the combination of full irrigation × RS-150 strain (4700 grains). The lowest GN (3648 grains) was recorded under deficit irrigation up to 50% podding × non-inoculation, although it did not differ significantly from the combination of deficit irrigation up to 50% flowering × non-inoculation (3721 grains).

According to the second-year data (Table 11), TGW was significantly influenced by irrigation regimes, showing a direct positive relationship with IR levels. The highest TGW (32.1 g) was obtained under full irrigation, which showed significant increases of 13.15% and 21.18% compared with deficit irrigation up to 50% flowering and up to 50% podding, respectively. Consistent with the first-year results, applying 100 kg N ha^− 1^ produced the highest TGW (31.64 g), which was significantly higher than those of the control (26.40 g) and 50 kg N ha^− 1^ (28.93 g) treatments, corresponding to increases of 19.85% and 9.37%, respectively. Furthermore, comparison of means related to the inoculation/non-inoculation factor indicated that the highest TGW (30.19 g) was recorded for seeds inoculated with the RS-153 strain, which was not significantly different from that obtained with RS-150 (29.76 g).

**Fig. 11:**
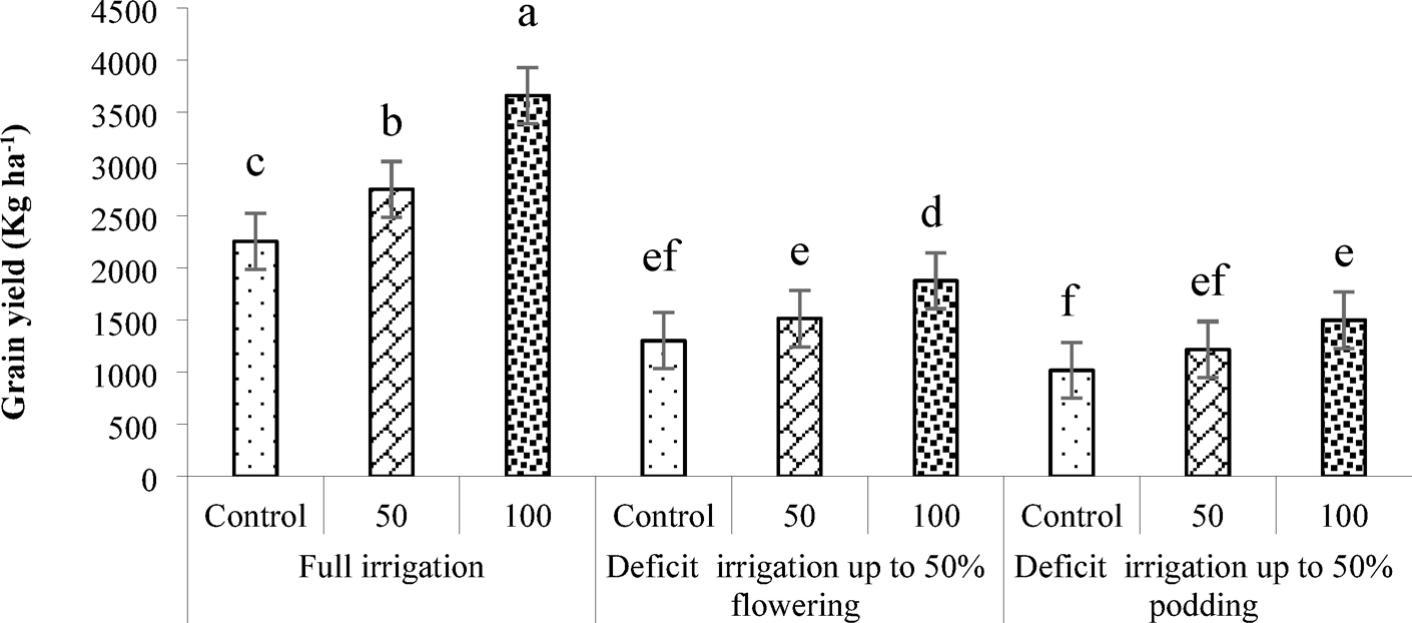
Interaction effects of) IR × NF levels on GN per square meter (adapted from the second-year data). Means followed by the same letter within each column do not differ significantly at *p* < 0.05.

Based on the second-year data (Fig. 11), comparing means related to the interaction effects of IR × NF on GY revealed that drought stress significantly reduced GY, although this reduction was partially compensated by N fertilizer application. Overall, the highest GY (3658.2 kg ha^−1^) was obtained from the interaction of full irrigation × 100 kg N ha^−1^, which showed a significant difference compared with other interaction treatments. In contrast, the lowest GY value (1017.8 kg ha^−1^) was recorded for the interaction of deficit irrigation up to 50% podding × non-application of NF. Overall, these results demonstrate that optimized water and nitrogen management, along with seed inoculation, can effectively moderate yield losses under drought stress, an aspect discussed in detail in the following section.

### Forage quality parameters

This section presents the effects of IR, NF levels, and BRh inoculation on key forage quality traits over two consecutive years (2021–2022 and 2022–2023). For clarity, comparisons focused on the best- and worst-performing treatments at each stage. The ANOVA results (Table 12) indicated that both IR and NF exerted significant effects (*p < 0.01*) on CrPro, ash%, ADF, DMD, and ME across both years, highlighting the critical role of water and nitrogen management in determining forage nutritive value. In contrast, BRh inoculation significantly influenced CrPro in the second year (*p < 0.01*), DMD in the first year (*p < 0.01*), ME in the first year (*p < 0.05*), and ash% in both years (*p < 0.01*). Notably, NDF was significantly affected by IR and the IR × NF interaction in the first year (*p < 0.01*) and by IR alone in the second year (*p < 0.01*). These findings suggest that, while water and nitrogen supply predominantly govern overall forage quality, microbial inoculation can further enhance specific traits, underscoring the potential of integrated management practices to optimize both yield and nutritive value of guar under varying environmental conditions.

**Table 12:** ANOVA (mean squares) for selected forage quality traits of guar under IR, NF levels, and *Bradyrhizobium japonicum* inoculation.

Source of variation	df	CrPr		NDF		ADF		Ash		DMD		ME	
		First year	Second year	First year	Second year	First year	Second year	First year	Second year	First year	Second year	First year	Second year
Block	2	1.05ns	3.88ns	10.3ns	12.1ns	18.21ns	0.43ns	1.33ns	1.09ns	10.96ns	0.99ns	0.32ns	0.03ns
Irrigation regimes (IR)	1	167.11**	233.48**	4266.5**	1025.3**	2158.22**	673.92**	59.2**	121.37**	1845.6**	724.69**	53.3**	20.98**
Ea	2	12.3	1.27	14.34	0.27	14.8	0.41	2.8	2.11	19.11	0.03	0.56	0.0009
Nitrogen fertilizer levels (NF)	1	48.85**	34.61**	0.99ns	13.2ns	113.92**	42.29**	65.34**	55.16**	128.2**	56.1**	3.69**	1.63**
*Bradyrhizobium (*BRh*)* inoculum	2	4.99ns	11.48**	0.67ns	0.14ns	38.54ns	1.03ns	18.01**	17.99**	34.2**	3.93ns	0.99*	0.11ns
IR × NF	1	19.8ns	0.0001ns	73.2**	5.33ns	0.29ns	0.19ns	0.47ns	1.06ns	1.38ns	0.13ns	0.04ns	0.004ns
IR × BRh	2	0.34ns	1.18ns	9.83ns	0.71ns	5.27ns	0.25ns	3.1ns	0.28ns	3.74ns	0.01ns	0.11ns	0.0004ns
NF × BRh	2	0.21ns	0.37ns	0.77ns	0.82ns	0.7ns	0.02ns	0.34ns	1.02ns	0.62ns	0.01ns	0.03ns	0.0003ns
IR × NF × BRh	2	1.65ns	27ns	5.35ns	0.08ns	0.89ns	0.11ns	1.11ns	0.37ns	0.13ns	0.09ns	0.004ns	0.002ns
Eb	25	5.06	1.64	14.62	8.99	13.97	3.17	2.81	2.92	9.59	2.06	0.28	0.06
CV		18.48	9.22	11.29	10.32	14.3	12.26	15.86	13.15	4.66	1.87	5.66	2.22

ns: non-significant; * and **: significant at *p* < 0.05 and *p* < 0.01, respectively.

According to Table 13, full irrigation and 100 kg N ha^− 1^ led to significant improvements in all forage quality parameters studied in both experimental years (except for ADF). Accordingly, full irrigation in both trial years resulted in notable increases of 43.01 and 44.89% for CPro, 21.02 and 15.53% for NA, 27.69 and 32.74% for ash, 24.16 and 12.45% for DMD, and 29.91 and 14.91% for ME, and significant decreases of 45.71 and 45.95% for ADF, respectively. Also, the application of 100 kg N ha^− 1^ in comparison to the control treatment significantly increased CrPro (18.44 and 15.27%), ash (29.28 and 21%), DMD (5.84 and 3.32%), and ME (7.13 and 3.98%), and decreased ADF (12.75 and 13.89%), respectively (Table 13).

**Table 13:** Mean comparisons (mean squares) of the effects of IR and NF levels on selected forage quality traits of guar.

Source of variation	CrPro (%)		ADF (%)		Ash (%)		DMD (%)		ME (MJ.Kg^− 1^ DM)	
	First year	Second year	First year	Second year	First year	Second year	First year	Second year	First year	Second year
IR										
Full irrigation	14.33a	16.43a	18.4b	10.2b	11.85a	14.88a	73.6a	81.12a	10.51a	11.79a
Deficit irrigation up to 50% podding	10.02b	11.34b	33.89a	18.87a	9.28b	11.21b	59.28b	72.14b	8.09b	10.26b
LSD	4.03	1.62	5.52	0.92	2.4	2.09	6.27	0.25	1.07	0.04
NF levels (kg ha^−1^)										
Control	11.01b	12.9b	27.92a	15.62a	9.22b	11.81b	64.56b	75.38b	8.98b	10.81b
100	13.34a	14.87a	24.36b	13.45b	11.92a	14.29a	68.33a	77.88a	9.62a	11.24a
LSD	1.56	0.89	2.6	1.24	1.17	1.19	2.15	0.99	0.37	0.17

Means followed by the same letter within each column do not differ significantly at *p* < 0.05.

**Fig. 12:**
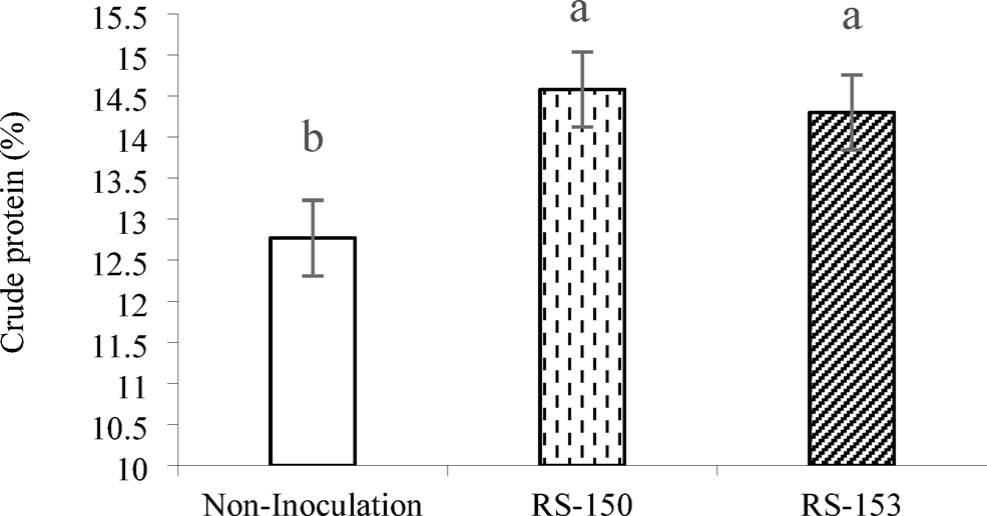
Effects of inoculation conditions on the CPro trait (Based on the first-year data). Means followed by the same letter within each column do not differ significantly at *p* < 0.05.

In addition, seed inoculation with *Bradyrhizobium japonicum* strains had a significant effect on CrPro values in the second year of the experiment. Under non-inoculated conditions, the CrPro value (12.77%) showed significant reductions of 12.41% and 10.70% compared to the values obtained from seeds inoculated with RS-150 and RS-153 strains, respectively (Fig. 12).

**Fig. 13:**
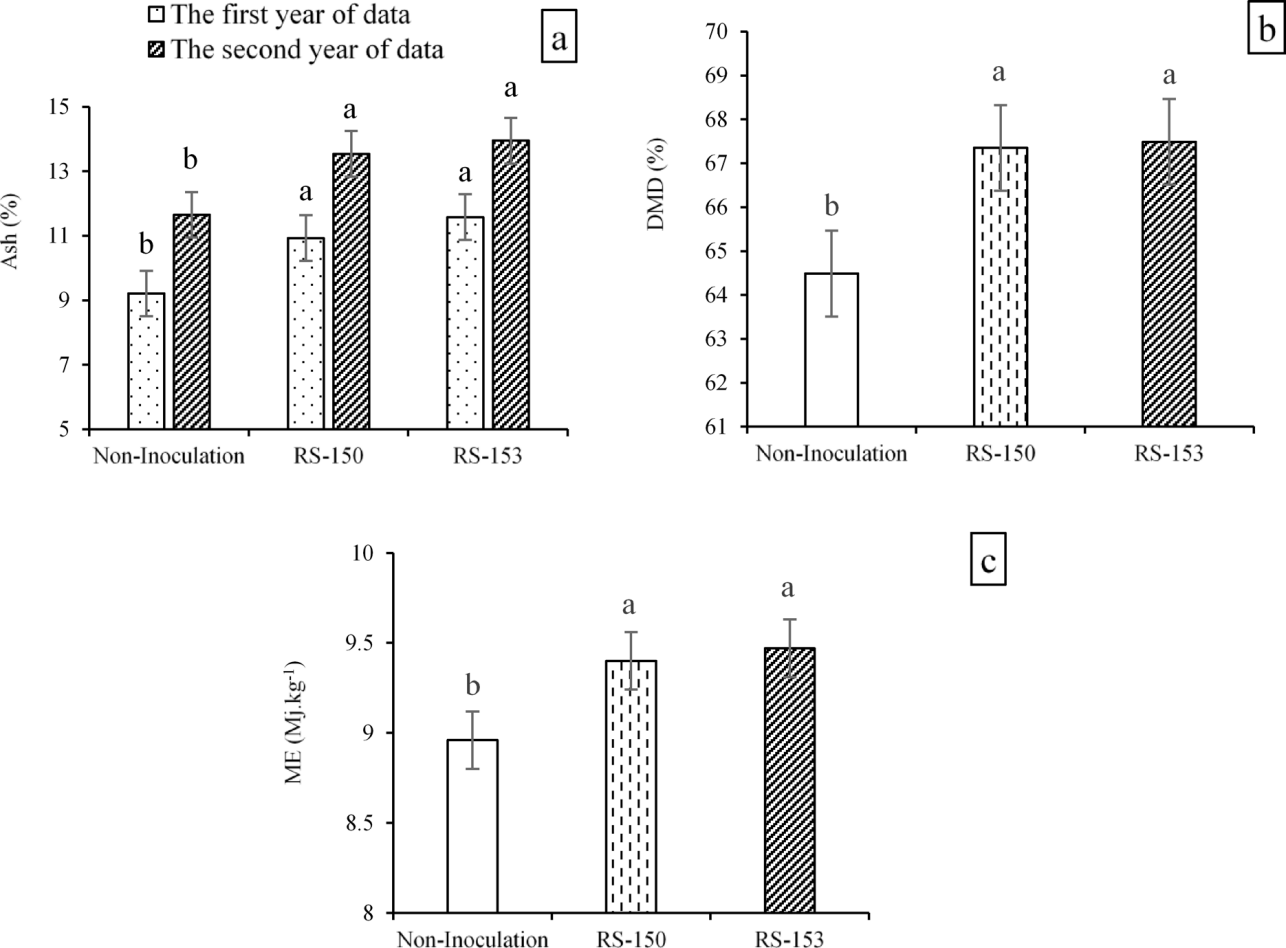
Effects of inoculation conditions on (**a**) Ash, (**b**) DMD, and (**c**) ME attributes. Means followed by the same letter within each column do not differ significantly at *p* < 0.05.

Based on the significant effects of the inoculation/non-inoculation factor on the ash percentage of guar plants in both years of the experiment, it was observed (Fig. 13a) that the highest ash percentages (11.85% in the first year and 13.95% in the second year) were obtained under the application of the RS-153 strain, which showed a significant difference compared to the control (non-inoculated) treatment. The lowest significant values of ash percentage were recorded in the control treatment (9.21% in the first year and 11.65% in the second year). In evaluating the effects of inoculation/non-inoculation treatments with different strains of *Bradyrhizobium* on DMD percentage in the first year of the experiment (Fig. 13b), the highest value of this trait (67.49%) was obtained from the application of the RS-150 strain, which showed no significant difference compared to the DMD value recorded under the RS-153 strain (67.35%). The lowest significant DMD value (64.49%) was observed in the non-inoculated (control) treatment. Finally, comparison of mean effects of inoculation conditions on ME percentage (adopted from first-year data; Fig. 11c) showed that non-inoculation of guar seeds caused significant decreases in ME. The ME values for the non-inoculated control, RS-150, and RS-153 inoculated treatments were estimated at 8.96, 9.40, and 9.47 MJ kg^−1^, respectively (Fig. 13c).

**Fig. 14:**
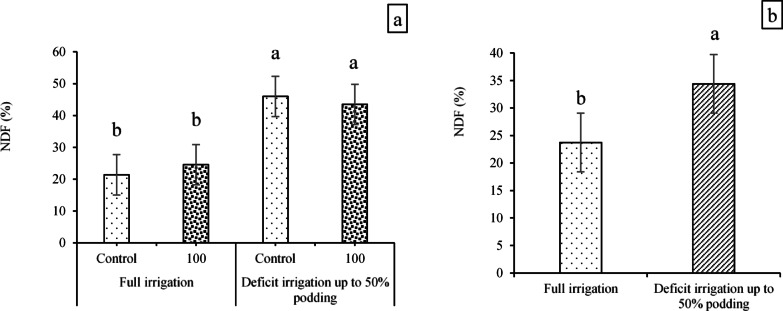
Effects (**a**) IR × NF interaction and (**b**) IR on NDF. Means followed by the same letter within each column do not differ significantly at *p* < 0.05.

Based on the first-year mean comparisons for the IR × NF interaction on NDF (Fig. 14a), the highest NDF value (46%) was observed under deficit irrigation up to 50% podding × no NF. In contrast, the lowest NDF values (37.21% and 36.24%) were recorded for full irrigation × no NF and full irrigation × 100 kg N ha^−1^, respectively. In the second year of the experiment (Fig. 14b), plants grown under full irrigation exhibited 23.73% NDF, which was 31.02% lower than those subjected to deficit irrigation up to 50% podding (34.4% NDF). These results highlight that both water availability and nitrogen supply strongly influence NDF accumulation, with water deficit substantially increasing fiber content, whereas full irrigation and adequate nitrogen reduce NDF and potentially improve forage digestibility.

## Discussion

As stated earlier, drought stress markedly altered phenological progression in guar, primarily through osmotic imbalance and reduced cellular turgor that constrain cell expansion and carbon assimilation^84,85^. This reduction in soil moisture not only shortens critical growth phases such as flowering (D50F), pod formation (D50P), and physiological maturity (DH), but also reflects a strategic shift in life-history traits, where the plant prioritizes early reproductive completion to escape severe stress. Rather than merely reflecting developmental acceleration, the shortened phenology observed under deficit irrigation suggests a strategic shift toward drought escape, where life-cycle completion precedes severe stress but at the expense of assimilate accumulation and sink development^86–90^. This pattern highlights a key adaptive mechanism in guar, emphasizing survival over maximal reproductive output, which has implications for timing irrigation and nutrient management to support yield stability. Similar stage-specific sensitivity has been reported across legumes, with reproductive stages exhibiting higher vulnerability due to their dependence on sustained carbon supply and translocation^91–93^. The observed reduction in phenological duration in the present study reflects a source/sink imbalance under water limitation, consistent with recent syntheses on drought-induced carbon partitioning trade-offs^94^. Although guar is recognized as a drought-adapted legume, its adaptive responses, such as stomatal regulation, reduced leaf expansion, and altered carbon allocation, primarily preserve survival rather than maximizing reproductive output^26,95–97^. Such mechanistic insights indicate that deficit irrigation management must consider phenological shifts to prevent critical yield losses.

Nitrogen supply significantly modulated plant responses to deficit irrigation. Adequate N availability sustains chlorophyll biosynthesis, Rubisco activity, and antioxidant metabolism, thereby maintaining photosynthetic efficiency under stress^98^. In this study, the application of 50–100 kg N ha^− 1^ not only prolonged flowering and podding phases but likely enhanced assimilate remobilization and reinforced reproductive sink strength, mitigating the source/sink imbalance induced by water limitation. This response aligns with findings that nitrogen enhances root proliferation, hydraulic conductance, and water uptake capacity, partially compensating for drought-induced reductions in turgor^99–101^. These interactions underline the synergistic potential of water and nitrogen management, suggesting that optimized N supply can maintain photosynthetic and reproductive function even under deficit irrigation. Importantly, water–nitrogen interactions exert a significant control on nitrogen use efficiency (NUE) under deficit irrigation; optimized nitrogen supply enhances nitrogen uptake and translocation, thereby preventing both dilution effects and metabolic limitation commonly observed under water stress. Such integrative management strategies may therefore improve both carbon and nitrogen use efficiency in water-limited systems, reinforcing yield stability. For instance, combined increases in irrigation and nitrogen markedly improved NUE components such as pre- and post-anthesis N translocation in wheat, indicating a synergistic effect of water and nitrogen on crop nitrogen dynamics^102,103^. Collectively, these findings emphasize the importance of synchronizing irrigation and nitrogen application to sustain both vegetative and reproductive functions under drought.

*Rhizobium* inoculation further reinforced drought resilience through multiple complementary mechanisms. Beyond biological nitrogen fixation, symbiotic bacteria enhance root architecture, exopolysaccharide production, and soil aggregation, improving rhizosphere water retention and nutrient availability^104–107^. PGPR-mediated hormonal modulation, particularly through the production and regulation of auxin (IAA) and cytokinins, stimulates root system growth and mitigates drought-induced senescence by modulating endogenous hormonal pathways controlling cell division, lateral root development, and stress signaling^84,108^. This integrated microbial effect likely underpins the observed improvements in chlorophyll, carotenoid content, and grain protein under combined N and *Rhizobium* treatments. These results suggest that integrating biological and mineral N sources enhances carbon allocation efficiency and strengthens reproductive sink establishment, providing a mechanistic explanation for improved yield components under water-limited conditions.

Biochemically, drought stress elevated soluble sugars and antioxidant enzyme activities (CAT, SOD), reflecting activation of osmotic adjustment and ROS detoxification pathways^109–111^. The accumulation of osmolytes and enhancement of enzymatic antioxidants stabilizes membranes, preserves photosystem II efficiency, and maintains cellular redox balance^112–116^. Under nitrogen fertilization and *Rhizobium* inoculation, these protective mechanisms were further strengthened, indicating that nutrient sufficiency supports metabolic resilience by sustaining both osmotic regulation and antioxidant capacity^117–119^. It should be noted that the observed decrease in CAT and SOD activities under higher nitrogen supply and *Rhizobium* inoculation likely reflects reduced oxidative stress resulting from improved water and nutrient status. In other words, the plants experience lower ROS accumulation and therefore require less enzymatic scavenging. This interpretation is consistent with the understanding that antioxidant enzyme activity is dynamic, responding to the intensity of oxidative challenge rather than serving as a static indicator of defense capacity. This interpretation is supported by evidence that antioxidant enzyme activities are dynamic and closely reflect the level of oxidative challenge; when ROS accumulation is reduced under improved water and nutrient status, the requirement for high CAT and SOD activity diminishes accordingly, indicating lower oxidative stress rather than impaired defense capacity, e.g., ROS have dual roles in plant stress signalling and antioxidant responses; both ROS production and antioxidant activity adjust dynamically to stress intensity^120,121^. From a functional perspective, these biochemical adjustments help maintain photosynthetic performance and partially protect yield formation under deficit irrigation.

Forage quality responses were closely aligned with physiological adjustments. Deficit irrigation increased fiber fractions (ADF, NDF) while reducing crude protein and digestible dry matter, consistent with stress-induced shifts toward structural carbohydrate accumulation^122–124^. This indicates that water limitation triggers a trade-off between structural integrity and forage nutritive value, which can influence livestock performance. In contrast, full irrigation combined with 100 kg N ha^− 1^ improved protein concentration, metabolizable energy, and digestibility, reflecting enhanced nitrogen assimilation and sustained carbon metabolism. Comparable irrigation–nitrogen interactions affecting forage nutritive value have been documented in annual ryegrass^125^. *Bradyrhizobium* inoculation further improved protein and digestibility parameters in a strain-dependent manner, consistent with enhanced N fixation and nutrient uptake efficiency^126–129^. These results collectively highlight that reproductive-stage water availability is critical for nutrient remobilization and forage quality, and that combined nutrient–microbial management can buffer quality losses under water stress.

A comparison with previous studies further strengthens the interpretation of the present findings. Consistent with our results, drought stress has been widely reported to reduce growth, seed yield, and forage quality in legumes, primarily through limitations in photosynthetic carbon assimilation and assimilate partitioning^130,131^, and in the present study, the observed reductions in phenological duration and forage quality under deficit irrigation are in line with these established responses. However, inoculation with *Bradyrhizobium* partially alleviated these constraints, which agrees with earlier reports demonstrating that rhizobial symbiosis enhances biological nitrogen fixation, improves root system architecture, and maintains plant water relations under drought conditions^84,132^. Similar enhancements in protein content and forage nutritive value following inoculation have been reported in legumes such as soybean and cowpea, where improved nitrogen availability and metabolic activity contributed to better yield quality under water-limited environments^105,133,134^. Furthermore, previous studies have shown that the combined application of deficit irrigation and plant growth-promoting rhizobacteria (PGPRs) leads to greater yield stability compared with non-inoculated treatments, highlighting the buffering role of plant–microbe interactions under stress conditions^135–137^. Nevertheless, some variability among studies has been noted, which may be attributed to differences in environmental conditions, soil fertility status, and rhizobial strain specificity^138,139^. In summary, the present findings corroborate existing evidence and further demonstrate that integrating microbial inoculation with optimized water management can effectively mitigate drought-induced limitations while sustaining both yield and forage quality in legume-based systems.

From a management perspective, integrated nitrogen and PGPRs are more effective to cope with drought conditions than single-factor interventions. Coordinated deficit irrigation with optimized nitrogen supply and symbiotic inoculation enhances physiological resilience, stabilizes sink development, and improves forage quality under semi-arid conditions. This integrative framework aligns with sustainable intensification principles highlighted by some researchers, where resource-use efficiency and stress buffering through coordinated water–nutrient management are central to productivity in water-limited agroecosystems^140,141^. Thus, these findings provide actionable guidance for optimizing irrigation scheduling, fertilization, and microbial inoculation to sustain both yield and forage quality in semi-arid legume systems.

Nevertheless, certain limitations should be acknowledged for this study. The study was conducted under specific environmental conditions and may not fully capture interannual climatic variability. Direct measurements of leaf water potential, gas exchange parameters, and nitrogen use efficiency indices were not included, limiting mechanistic quantification. Future research integrating physiological, isotopic, and molecular analyses would allow more precise causal inference regarding water, nitrogen, and PGPRs interactions and improve the transferability of results across diverse agroecosystems. Overall, the results demonstrate that deficit irrigation primarily constrains guar productivity through phenological shortening, source/sink imbalance, and oxidative stress, whereas optimized nitrogen fertilization and *Rhizobium* inoculation mitigate these constraints by sustaining photosynthetic capacity, antioxidant defense, and reproductive allocation. The synergistic integration of irrigation scheduling, nitrogen management, and microbial inoculation therefore represents a robust strategy for stabilizing guar yield and forage quality under arid and semi-arid conditions, providing both mechanistic insight and practical guidance for water-limited agroecosystems.

## Conclusion

This study demonstrated that drought stress significantly reduced phenological duration, disrupted assimilate partitioning, and impaired grain development in guar, leading to decreased yield and forage quality. Shortened flowering and pod-filling phases under water deficit limited the accumulation of storage compounds, while osmotic imbalance and oxidative stress further constrained plant growth. Conversely, nitrogen fertilization enhanced chlorophyll content, photosynthetic efficiency, and protein synthesis, providing additional resources for seed filling and forage quality. Inoculation with *Bradyrhizobium* strains improved symbiotic nitrogen fixation and nutrient assimilation, contributing to higher crude protein content and improved forage digestibility. The combined application of nitrogen and *Bradyrhizobium* effectively mitigated drought effects by promoting osmotic adjustment, activating antioxidant defenses, and facilitating translocation of photosynthates to developing seeds. These findings highlight that integrating optimized irrigation scheduling with balanced nitrogen fertilization and effective rhizobial inoculation is a practical strategy to maintain yield and forage nutritive value under limited water availability. Future research should validate these findings over multiple growing seasons and investigate genotype-specific responses to refine sustainable management strategies for guar cultivation.

## Data Availability

The datasets generated, used, deployed, and analyzed during the present study are not accessible to the general public. However, they are available from the corresponding author upon adequate request.

## Abbreviations

N: Nitrogen
BRh: Bradyrhizobium inoculum
D50F: Days to 50% flowering
D50P: Days to 50% podding
DH: Days to harvesting
CAT: Catalase
GN: Grain number
TGW: 1000-grain weight
GY: Grain yield
Chl: Chlorophyll
CAR: Carotenoid
GPr: Grain protein
SS: Soluble sugars
PC: Proline content
CrPr: Crude protein
ADF: Acid detergent fiber
NDF: Neutral detergent fiber
DMD: Dry matter digestibility
ME: Metabolizable energy
IR: Irrigation regimes
NA: Nitrogen absorption
NF: Nitrogen fertilizer
FW: Fresh weight
DW: Dry weight
SOD: Superoxide dismutase
CV: Coefficient of variation

## Declarations

### Declaration of compliance

We confirm and ensure that the study follows all relevant guidelines and regulations and complies with the IUCN Policy Statement on Research Involving Species at Risk of Extinction and the Convention on the Trade in Endangered Species of Wild Fauna and Flora.
